# Building a DNA Barcode Reference Library for the True Butterflies (Lepidoptera) of Peninsula Malaysia: What about the Subspecies?

**DOI:** 10.1371/journal.pone.0079969

**Published:** 2013-11-25

**Authors:** John-James Wilson, Kong-Wah Sing, Mohd Sofian-Azirun

**Affiliations:** 1 Museum of Zoology, Institute of Biological Sciences, Faculty of Science, University of Malaya, Kuala Lumpur, Malaysia; 2 Institute of Biological Sciences, Faculty of Science, University of Malaya, Kuala Lumpur, Malaysia; University of Guelph, Canada

## Abstract

The objective of this study was to build a DNA barcode reference library for the true butterflies of Peninsula Malaysia and assess the value of attaching subspecies names to DNA barcode records. A new DNA barcode library was constructed with butterflies from the Museum of Zoology, University of Malaya collection. The library was analysed in conjunction with publicly available DNA barcodes from other Asia-Pacific localities to test the ability of the DNA barcodes to discriminate species and subspecies. Analyses confirmed the capacity of the new DNA barcode reference library to distinguish the vast majority of species (92%) and revealed that most subspecies possessed unique DNA barcodes (84%). In some cases conspecific subspecies exhibited genetic distances between their DNA barcodes that are typically seen between species, and these were often taxa that have previously been regarded as full species. Subspecies designations as shorthand for geographically and morphologically differentiated groups provide a useful heuristic for assessing how such groups correlate with clustering patterns of DNA barcodes, especially as the number of DNA barcodes per species in reference libraries increases. Our study demonstrates the value in attaching subspecies names to DNA barcode records as they can reveal a history of taxonomic concepts and expose important units of biodiversity.

## Introduction

Surveys of butterfly species have often been considered good surrogates for surveys of total biodiversity (e.g. in Malaysia [1]). This is because of their role in food webs - caterpillars consume large quantities of plants and are themselves consumed by other animals in large numbers - and because, relative to most other animal groups, collecting and identifying adult butterflies is considered easy [1]. This is particularly so in Peninsula Malaysia where butterflies have received intensive taxonomic attention. The “Butterflies of the Malay Peninsula” have been the subject of a series of comprehensive field guides, beginning with Distant in 1882–1886 [2], and followed by four editions of Corbet and Pendlebury’s classic checklist, first published in 1934 [3] and most recently revised by Eliot in 1992 [4]. Butterflies have benefitted and suffered from intensive taxonomic attention. In many cases a preponderance of names exists for the same species and names are often used incorrectly (see list of synonyms in [4]). During a recent survey of butterflies in Southern Thailand, 150 km north of the Malaysian border, fewer than 50% of the observed butterflies were identified to species [5]. Adding to these difficulties is widespread but inconsistent use of butterfly subspecies names and concepts [6]–[7]. Butterfly surveys in Peninsula Malaysia have not been consistent in using or ignoring subspecies names [8]–[10]. This can make a big difference to biodiversity surveys - if we consider species as the biodiversity unit there are 793 units in Peninsula Malaysia, but if subspecies is considered the biodiversity unit, the number rises to 930 [11].

Butterfly trinomials have traditionally been used to recognize ‘moderate’ morphological differentiation correlated with disjunct geographical distributions [6]–[7], [12]. However, non-discrete morphological variation and the application to contiguously distributed populations, often make subspecies boundaries ambiguous [7]. Following Tobias et al. [13]’s recommendations for avian subspecies delimitation, Braby et al. [7] recently suggested standardized phenotypic criteria for subspecies delimitation in butterflies. Although considered desirable, Braby et al. [7] refrained from setting criteria based on DNA characters, citing a lack of data. However, they did acknowledge that under their concept, subspecies are genetically distinct, but not reciprocally monophyletic according to mitochondrial DNA, noting that lineages possessing a diagnostic morphological character and also showing reciprocal monophyly are probably better regarded as distinct species [7]. This criterion of concordance for species delimitation is in line with “state-of-the-art” practice in taxonomy i.e. the MTMC (Mitochondrial Tree Morphological Character congruence) of Miralles and Vences [14].

Mitochondrial DNA barcodes [15]–[16] are increasingly being used as a supplementary taxonomic identification tool in surveys of Lepidoptera (e.g. [5], [17]–[19]). However, DNA barcoders have often ignored subspecies names [18], [20]–[21], and have used personalized alphanumeric codes for biodiversity units discovered below the traditionally recognized species boundary (e.g. *Hamadryas* feroniaECO01 [18], [22]). These units used to account for previously overlooked (and possibly cryptic) diversity have come to be known as “dark taxa” [23] and the correspondence between subspecies, recognized by morphological differentiation, and dark taxa is often difficult to resolve (e.g. does *H.* feroniaECO01 = *H. feronia farinulenta*? [22]). Most GenBank [24] and BOLD [25] records do not include subspecies names, meaning it is impossible to tell if the authors of the DNA sequence could determine which subspecies the butterfly belonged to or not. It may be possible to narrow down subspecies identity based on locality, but locality is often missing, or imprecise, for GenBank records too.

The aim of this study was to build a DNA barcode reference library for the true butterflies (species from the families – Papilionidae, Pieridae, Nymphalidae, Lycaenidae, Riodinidae) of Peninsula Malaysia from specimens in the Museum of Zoology, University of Malaya (UMKL) collection. We tested the capacity of the library to function as an accurate identification tool for species, screening for signatures of misidentifications, of multiple species sharing identical or very similar DNA barcodes, and of currently unrecognised diversity within the collection. Given the inconsistency in using or ignoring subspecies names in surveys of butterflies, we also explored the value of attaching subspecies names to records in DNA barcode reference libraries. The new DNA barcode library for Peninsula Malaysia was analysed in conjunction with publicly available DNA barcodes from other Asia-Pacific localities to test the ability of the DNA barcodes to discriminate subspecies. Are butterfly subspecies distinctive biodiversity units that can be distinguished by their DNA barcodes and if so, what differentiates them from species? This is an important question. Twenty-eight native butterfly species are currently protected under Malaysian law [26] but in other jurisdictions subspecies can also have legal status [27].

## Materials and Methods

### Building a DNA Barcode Reference Library for the True Butterflies of Peninsula Malaysia

The UMKL butterfly collection comprises three thousand specimens with representatives of around 30% of the known fauna of Peninsula Malaysia. DNA barcodes were obtained by sampling dry legs from specimens in UMKL. Sampling was restricted to a few specimens per species, including morphologically and geographically diverse specimens where possible. Taxonomy and nomenclature follows our scratchpad [11], (see [28]) and reflects taxonomic decisions since Eliot [4]. The legs were sent to the Canadian Centre for DNA Barcoding for DNA barcode assembly following standard high-throughput protocols for insects [29]. Details of the specimens and DNA barcodes (including GenBank accessions) are available on BOLD [25] in the public dataset: DS-BUTMAY and in Table S1.

We performed an initial screen of the dataset by blasting each new DNA barcode against the full database of BOLD. In cases where new DNA barcodes matched DNA barcodes assigned to a different species name (with >98% similarity) we reexamined the specimens’ morphology to determine the accuracy of the original identifications (provided in the “Taxonomy Note” field of the specimen records on BOLD).

Following this initial screen we subsequently noted cases where specimens currently with different species names have identical or similar DNA barcodes (with >98% similarity) and cases where specimens currently with the same species name have dissimilar DNA barcodes (≤98% similarity). The genetic distances referred to are all K2P corrected (Kimura 2-parameter; as provided by BOLD). We used 2% as the basis for our screening following the example of previous DNA barcoding studies (e.g. [15], [17], [21]–[22], [30]) which have demonstrated that although there is no expectation for a universal threshold of genetic distances between or within species, 2% provides a useful heuristic upon which to base deeper investigation.

### Testing if Subspecies can be Distinguished by their DNA Barcodes

**Figure 1:**
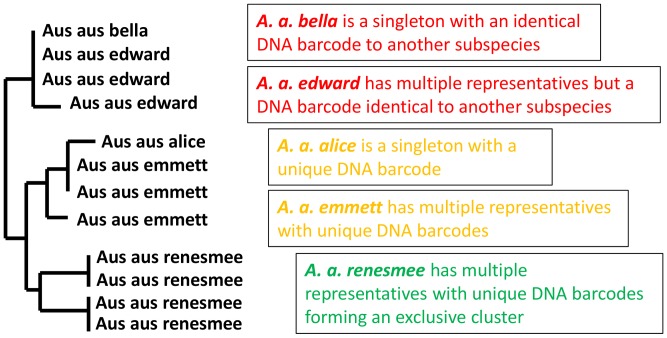
Criteria for determining subspecies distinctiveness on Neighbor-joining trees.

By blasting the UMKL DNA barcodes against the full BOLD database we determined which species in the dataset have DNA barcodes on BOLD from other researchers (see Table S1). When a subspecies name was not provided we derived a subspecies name for these DNA barcodes by searching published accounts of the DNA sequences (i.e. journal articles or authors’ websites, e.g. [31]–[32]) and by making inferences based on the reported geographical distribution of the subspecies (e.g. [33], [34]). Note that many DNA barcodes come from GenBank with poorly reliable data, especially imprecise geographical origin, or are “Private” or “Early Release” on BOLD and not publicly viewable, but which nevertheless contribute to a BOLD identification. Where a species from UMKL was determined to be present on BOLD with DNA barcodes from multiple subspecies we then examined if the subspecies were distinguishable based on a “Tree Based Identification” (Neighbor-Joining) in BOLD (see Subspecies Trees S1). Specifically, we observed if each subspecies: i) shared identical DNA barcodes with another subspecies; ii) had unique DNA barcodes but which did not form an exclusive cluster on the tree provided by BOLD; iii) had unique DNA barcodes which formed an exclusive cluster (Figure 1).

## Results and Discussion

### A DNA Barcode Reference Library for Identification of True Butterflies in Peninsula Malaysia

A DNA barcode was obtained from 458 of 561 specimens (82%) submitted for analysis, accounting for 233 species. While similar to that reported for other Lepidoptera DNA barcoding studies (e.g. [17], [35]), considering that the oldest specimen submitted for analysis was 20 years old the success rate seemed low for a relatively recent collection. This could serve as a warning for those attempting to build a DNA barcode library from tropical museum collections (but see [18]) and has prompted a review of specimen storage conditions at UMKL. An approach that has been suggested is to freeze newly collected butterflies and store them as frozen tissue vouchers rather than the traditional pinning and drying of specimens. DNA extraction, amplification and sequencing using ‘Lep’ primers [29] was highly efficient with fresh (<3 yrs) material. Further mining of public and private collections coupled with targeted field sampling should gradually move the library to completion and increase the number of representatives per species. However, in view of the hyper-diversity of Peninsula Malaysia [4] this is a challenge compared to temperate regions (e.g. the 180 butterfly species of Romania [11]).

Screening the new DNA barcode dataset against the full BOLD database followed by reexamination of morphology revealed that about 15% of specimens in the UMKL collection were originally misidentified. Many of these were nymphalids from the subfamily Satyrinae and the tribe Heliconiini within the Heliconiinae. One noteworthy case was a pierid originally identified as *Delias barcasa dives* and collected at Genting Highlands, Pahang, in 2011. DNA barcoding conclusively assigned the specimen to *Delias agostina* (99.3% similarity with DQ082779 from Chiang Mai in northern Thailand [36]) confirming the presence of the species in Peninsula Malaysia. *Delias agostina* is not included in the plates of D’Abrera [4] but is featured in the Corbet and Pendlebury *Delias* key with “Burma” printed in bold and in the species checklist with an asterisk, indicating resident status as unconfirmed [4]. Successive screening also revealed several cases of multiple species within the same genus showing identical or similar DNA barcodes.

UMKL DNA barcodes for *Danaus melanippus hegesippus* shared 99.1% similarity with a “Private” *D. genutia* DNA barcode from Australia (subspecies not given but probably *D. g. alexis* [34]) which in turn was >2% distant from UMKL *D. g. intermedius* DNA barcodes. The Australian subspecies has previously been treated as a distinct species [34]. Interestingly, the phylogenetic sister of *D. melanippus, D. affinis* (according to [37]), was not the closest matching species, being >2.9% from *D. melanippus* and >2.6% from *D. genutia*.

UMKL DNA barcodes recorded under *Euploea modesta modesta* matched closely (<99.8%) with GenBank DNA barcodes from India recorded under *E. core* [38] and “Early-Release” DNA barcodes (98.8%) recorded under *E. alcathoe* and *E. core* from Australia and Papua New Guinea (Euploea Tree S1). *E. m. modesta* is found in India, *E. m. lugens* in Australia and Papua New Guinea. Similarly, the single short UMKL DNA barcode (307 bp) for *E. camaralzeman malayica* matched closely (99.6%) with a “Published” DNA barcode for *E. core* from Thailand and matched 100% to other “Early-Release” *E. core* DNA barcodes on BOLD. Furthermore, the UMKL DNA barcodes for *E. doubledayi evalina* matched 100% to *E. algea* (KC306717) from India and yet another “Early-Release” *E. core* from Australia. There was a further distinct cluster of *E. core* on BOLD containing DNA barcodes from Australia and Thailand which was distant from all the UMKL *Euploea*. One UMKL DNA barcode recorded under *Euploea eunice leucogonis* and collected from Genting Highlands, Pahang, in 2012 was distant (3.3%) from the two other UMKL *E. eunice leucogonis* DNA barcodes (Euploea Tree S1), which themselves were similar (99.2%) to *E. kluji* from India (KC306728) but relatively distant (98.0%) from *E. kluji* from Southern Thailand (HQ962260). The morphologically similar, and one time synonym [4], *E. leucostictos* formed a distinct sister to this cluster. As wittily noted by Corbet and Pendlebury (2nd edition) in the legend to Plate 23 [39], “it is easier to ascertain the country of origin of a (*Euploea*) specimen than to determine its specific identity”, the genus is notorious for being taxonomically difficult. Any taxonomic interpretation is further complicated by reports of hybrids [40] and the fact that species are commonly reared for butterfly parks (and released). There may be a tendency for collectors to assign difficult specimens to the most common species - *E. core* - accounting for its appearance in many places in this screening.

Identification of *Eurema* species, a genus found abundantly in disturbed and undisturbed habitats alike, is also notoriously difficult [4], [41]. UMKL DNA barcodes recorded under three species of *Eurema* (*E. ada iona*, *E. hecabe contubernalis*, *E. lacteola lacteola)* showed low divergence amongst themselves and also with various *Eurema* species from various Asia-Pacific localities. The DNA barcodes all sat within the same BIN (Barcode Index Number) (BIN S1); the system on BOLD which clusters DNA barcodes into operational taxonomic units closely corresponding to traditionally recognized species [42]. A review of *Eurema* in Peninsula Malaysia is currently underway by our research group. Whether *Eurema* as an example of ‘barcode sharing’ is actually a reflection of the difficulty assigning these small yellow butterflies to species on the basis of wing patterns remains to be seen.

*Loxura atymnus fuconius* and *L. cassiopea cassiopea* are morphologically similar [4] and the UMKL specimen of *L. atymnus fuconius* was originally recorded under *L. cassiopea cassiopea*. However, these species cannot be confused as the wing patterns, when studied carefully, and the DNA barcodes, although close (1.7% distant and in the same BIN), are characteristic for each species.

**Figure 2:**
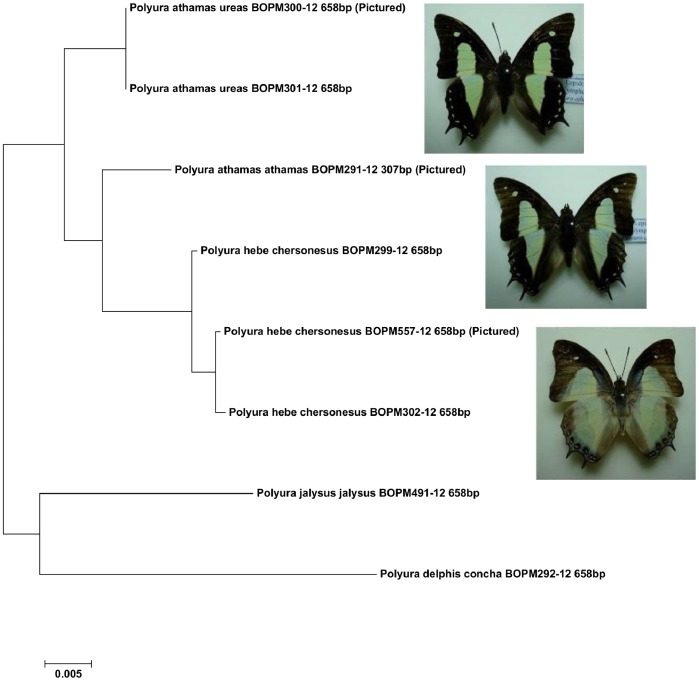
Neighbor-joining tree showing the K2P distances between *Polyura* DNA barcodes. The BOLD Process ID is followed by the sequence length.

In the UMKL dataset the single representative of *Polyura athamas athamas* was distant from the *P. a. uraeus* DNA barcodes (2.1%) and closer to *P. hebe* (1.7%). Like Eliot [4] we are hesitant to draw conclusions about the species status of these two taxa, in our case because of the small number of specimens available in UMKL and because only a short DNA barcode (307 bp) was generated for the *P. a. athamas* specimen. However, these taxa are easily distinguished as the wing patterns and the DNA barcodes, although close, are characteristic for each taxon (Figure 2).

UMKL DNA barcodes recorded under four species of *Tanaecia* (*T. aruna aruna*, *T. iapis puseda*, *T. munda waterstradti*, *T. pelea pelea*) sat in the same BIN along with three DNA barcodes from Thailand, also representing multiple species (BIN S2). The taxonomy of this genus is difficult [43], with species specific diagnostic characters mostly from the male genitalia [4], [43] (not studied here), and needs further investigation.

**Figure 3:**
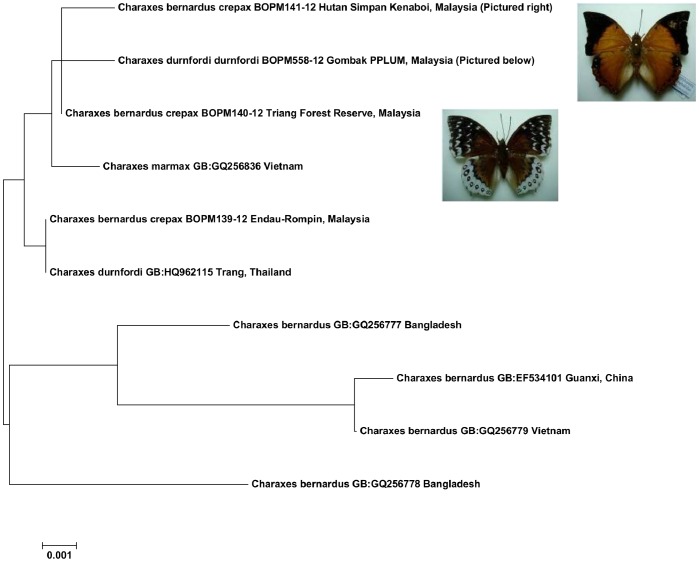
Neighbor-joining tree showing ‘DNA barcode sharing’ in the genus *Charaxes*. The BOLD Process ID or GenBank Accession (GB) is followed by locality.

Non-monophyly of *Charaxes bernardus* has been reported before, with *C. marmax* nested within *C. bernardus* on the molecular phylogenetic tree of Aduse-Poku et al. [44]. We found that *C. durnfordi durnfordi* and *C. bernardus crepax* shared identical DNA barcodes, despite very distinctive wing patterns (Figure 3). This interesting and rare pattern deserves further study and may reflect the complex biogeographical history of this genus [44] or mitochondrial introgression.

UMKL DNA barcodes recorded as *Mycalesis mineus macromalayana* sat in a BIN with GenBank DNA barcodes for *M. mineus* from India, but the BIN also contained DNA barcodes from GenBank recorded under *M. visala*, *M. intermedia* and *M. perseoides* (BIN S3). Also present were unpublished *M. mineus* and *M. panthaka* DNA barcodes from China. Like the other genera above the Malaysian *Mycalesis* have a long history of taxonomic difficulty [45].

UMKL DNA barcodes recorded as *Tirumala septentrionis septentrionis*, the only common *Tirumala* in Peninsula Malaysia, sat in a BIN with “Early Release” *T. hamata* DNA barcodes from Australia and Papua New Guinea [35] (BIN S4). *T. septentrionis septentrionis* overwintering in Taiwan has previously been treated as *T. hamata septentrionis* [46]. *T. limniace*, a similar looking species, DNA barcodes from India were also in the BIN and may be misidentifications.

UMKL *Troides helena cerberus* DNA barcodes matched closely (>98.8%) with GenBank and BOLD *T. oblongomaculatus* from Indonesia. These closely related species have been treated historically as a single species [34]. *T. oblongomaculatus,* a “relic race of uncertain status” [47], has been reported to hybridize, including with taxonomically distant species [48].

UMKL DNA barcodes recorded under *Ypthima horsfieldi humei* shared close similarity (>99.5%) with *Ypthima nebulosa* DNA barcodes from Thailand [5]. *Y. nebulosa* has not been reported for Peninsula Malaysia [34] but according to Corbet and Pendlebury is likely to be found in the region [4] suggesting the specimens in UMKL require further evaluation.

**Figure 4:**
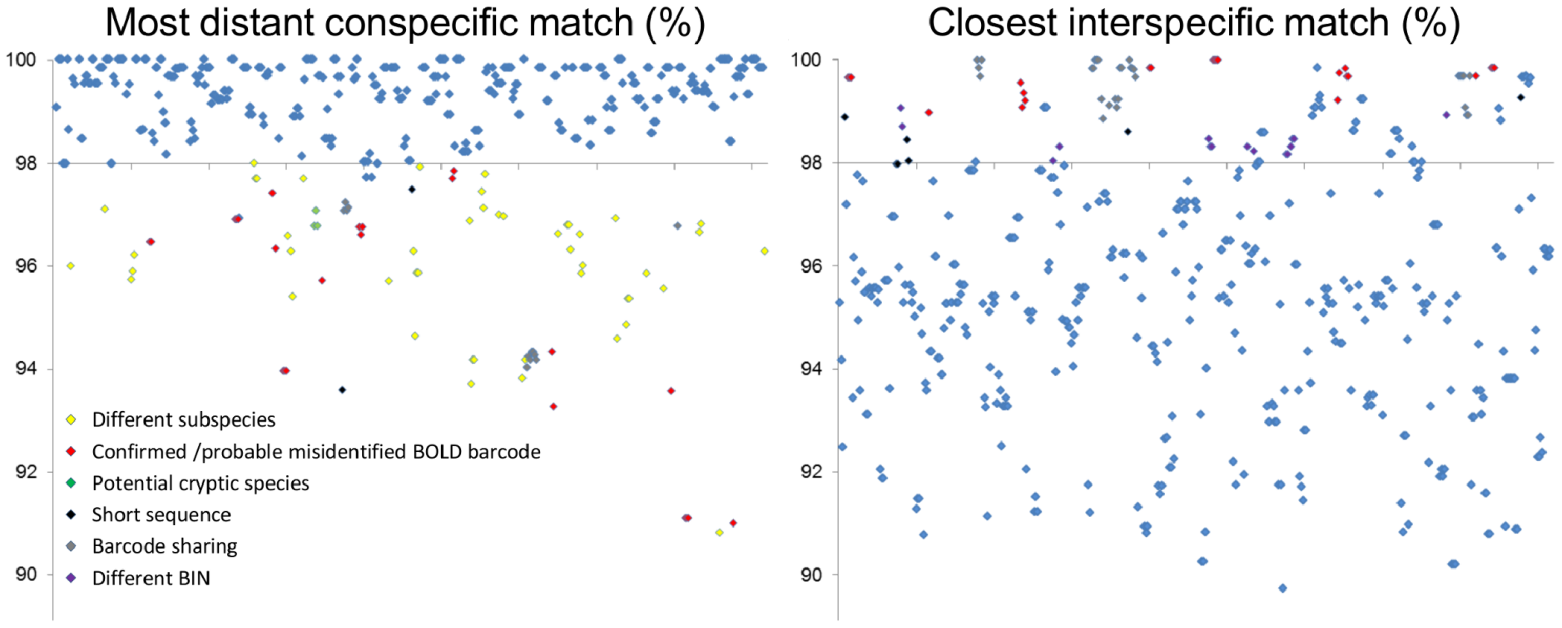
Most distant conspecific and closest interspecific matches for 458 UMKL DNA barcodes when blasted against the full BOLD database. The DNA barcodes are arranged alphabetically by species name along the horizontal axes. Conspecific similarities below 98% and interspecific similarities above 98% that were associated with different subspecies, misidentified BOLD barcodes, potential cryptic species, short sequence length, barcode sharing and different BINs are highlighted with different coloured data points.

Screening against BOLD highlighted 27 other species with unique DNA barcodes but which were <2% distant from other species. These represented borderline cases for the screening threshold which were nevertheless allocated to different BINs by BOLD (see Table S1; Figure 4) and cases associated with short sequence lengths or suspected misidentified DNA barcodes on GenBank/BOLD (see Table S1; Figure 4).

**Figure 5:**
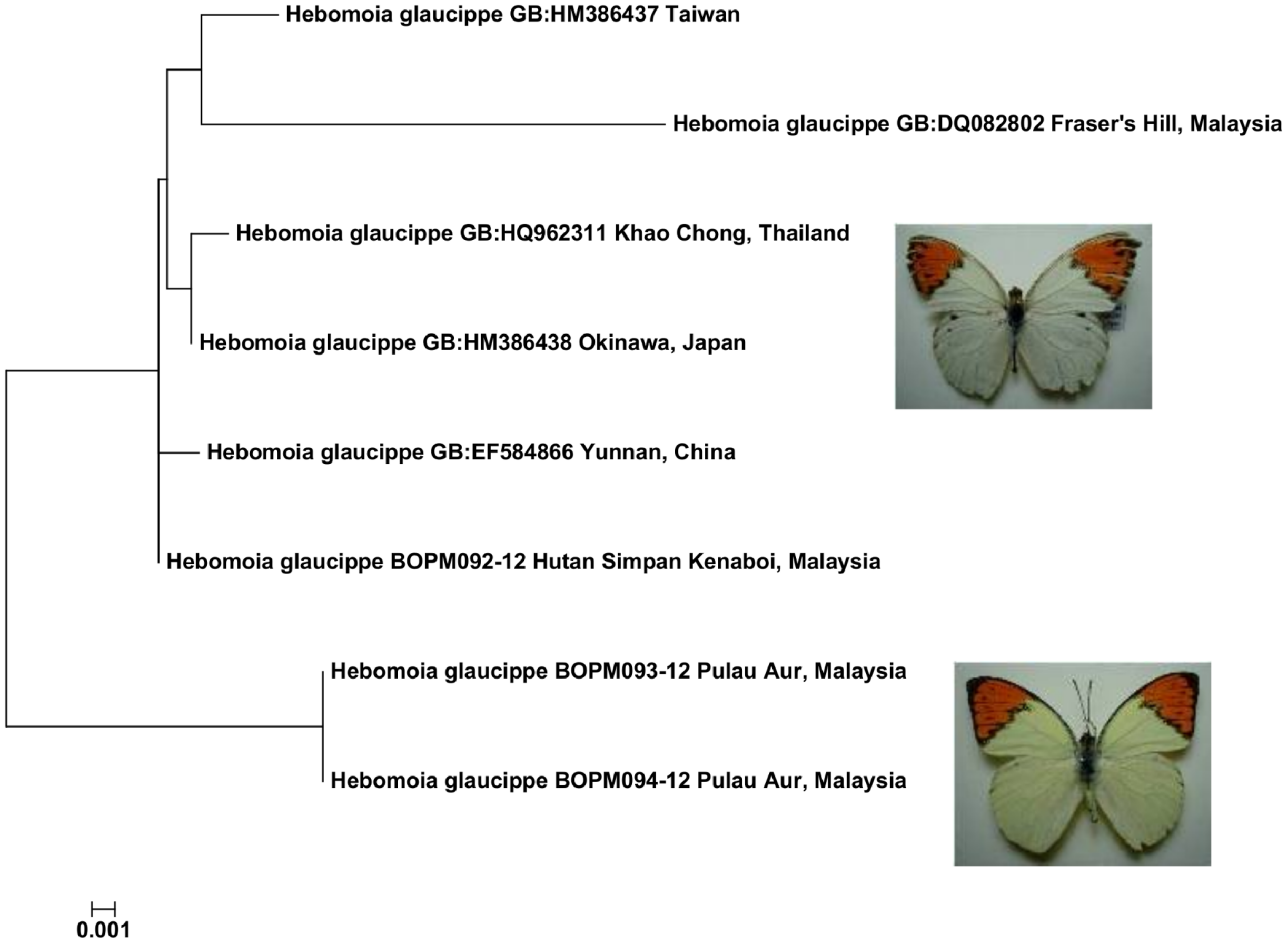
Neighbor-joining tree showing K2P distances between *Hebomoia glaucippe* DNA barcodes. The BOLD Process ID or GenBank Accession (GB) is followed by locality.

Within the new Peninsula Malaysia dataset, only three species showed wide (>2%) conspecific distances: *Euploea eunice*, *Polyura athamas* (see above) and *Hebomoia glaucippe*. DNA barcodes for *H. g. anomala* found on Pulau Aur, Johor, a small island off the east coast of mainland Peninsula Malaysia, were 4.2% distant from the DNA barcode for *H. g. aturia* from the mainland which clustered closely with BOLD DNA barcodes from Thailand, most likely *H. g. aturia*, and different subspecies from Taiwan and China (Figure 5). The differences in wing pattern between these two groups are readily apparent with the Pulau Aur butterflies exhibiting a deeper yellow upperside [4] (Figure 5). *H. g. anomala* was described as a distinct species by Pendlebury in 1939 [34].

Compared with the levels of cryptic diversity discovered in other DNA barcoding surveys (e.g. [18], [22], [49]) three species showing wide conspecific distances is relatively few, suggesting that the long history of taxonomic study of the butterflies of Peninsula Malaysia has led to a relatively accurate account of species diversity. Furthermore, two of the three cases, *Polyura* and *Hebomoia*, were associated with subspecies, one of which had previously been treated as distinct species. There were no other cases of species being represented by more than one subspecies in the UMKL collection. Perhaps the one case of truly unrecognized diversity within the Peninsula Malaysia dataset was the distinct DNA barcodes within *Euploea eunice leucogonis* and this deserves further study to determine if this is truly the exception.

Following correction of morphological misidentifications in UMKL, the DNA barcodes for 78% of the 233 species were unique (with non-overlapping conspecific and interspecific distances for multiple representatives) when compared with conspecifics and closest matches on BOLD (Table S1; Figure 4). Excluding outliers - confirmed or probable misidentified DNA barcodes on BOLD and conspecific distances associated with divergent subspecies or cryptic species diversity - the number of distinct species rises to 92%, validating the capacity of the DNA barcode reference library for rapid and effective assignment of true butterflies to species. The few cases of ‘barcode sharing’ that remain provide stimulus for subsequent studies. Considering the importance of butterflies as bioindicators and conservation flagships we are particularly encouraged by the potential of DNA barcoding to enable local species inventories, without the need for lethal sampling [50], but with much higher accuracy and precision than can be achieved by observing butterflies on the wing [1], [5], or even by traditional morphological identification (considering the misidentifications in UMKL).

### Can Subspecies be Distinguished by their DNA Barcodes and What Differentiates them from Species?

**Figure 6:**
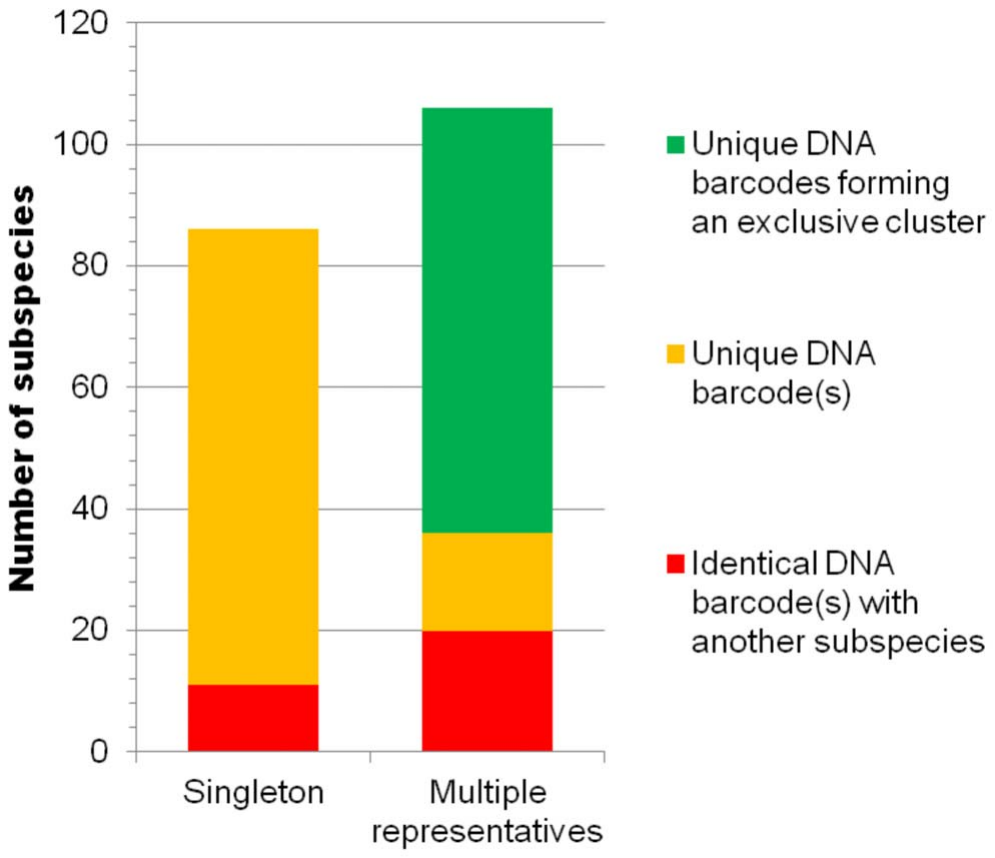
Distinctiveness of DNA barcodes from 192 subspecies, representing 80 species of true butterflies.

There were 1189 DNA barcodes on BOLD for the 233 UMKL species and we determined that 80 species were represented by multiple subspecies (Table S1). Of the 192 subspecies, 86 were represented by singletons and 87% of these singletons had unique DNA barcodes. Of the 106 subspecies represented by multiple DNA barcodes 81% had unique DNA barcodes not shared with other subspecies and 66% formed exclusive clusters on identification trees (Subspecies Trees S1; Figure 6). Because many of the subspecies were represented by singletons and “Early-Release” or “Private” DNA barcodes on BOLD, it is outside the scope of this study to examine how many of the subspecies would be reciprocally monophyletic for mtDNA in phylogenetic (maximum or statistical parsimony) analyses. However, under current levels of representation, most subspecies are genetically distinct for mtDNA (Figure 6) which is in accordance with the expectations of the butterfly subspecies concept of Brady et al. [7]. How this pattern changes or stabilizes as BOLD continues to grow will clarify the nature of the relationship between DNA barcodes and subspecies more accurately. The results suggest that as subspecies move from singletons to multiple representatives the number of subspecies with unique DNA barcodes could decrease (87% versus 81%; Figure 6). Because the butterfly DNA barcodes available on BOLD came from a range of local surveys or phylogenetic studies, the geographic coverage was patchy and no biogeographic patterns were apparent from the analysis. However, it was not uncommon for UMKL DNA barcodes to be similar to conspecific DNA barcodes from India or China, at the extremities of the Asia-Pacific region while distinct subspecies were from one of the region’s many islands.

Of the subspecies with unique DNA barcodes many were highlighted when we screened the UMKL DNA barcode dataset against the full BOLD database using the >2% conspecific distance threshold (Table 1). Conspecific genetic distances of this magnitude, i.e. distances typically seen between species, would normally warrant “dark taxon” status in DNA barcoding studies and some of these cases have in fact been highlighted by previous studies (e.g. several species from Western Ghats, India [39]). Historical studies have likewise highlighted the morphological distinctiveness of these taxa as implied through their current disparate subspecies designations. Many of these subspecies had previously been treated as distinct species (Table 1), and the DNA barcode data supports a re-evaluation of their status. Similarly, ‘unrecognized’ lepidopteran diversity revealed through DNA barcoding in the other surveys (e.g. [18], [22]) had been recognized previously, although as subspecies taxa, or as sunken or forgotten names [51]. This may reflect the challenge of meshing Linnaean taxonomy with DNA taxonomy systems [52]. In these cases above, consistent application of subspecies names in DNA barcode reference libraries would negate the need for dark taxon designation. Following a reverse MTMC [14], DNA barcoding could provide a means of testing, through concordance, if subspecies, established on the basis of moderate morphological differentiation between localities [7], are of sufficient evolutionary independence to merit species status. Note that 13 other species had specimens with unique DNA barcodes but which were >2% distant from conspecific DNA barcodes. However, these further cases were due to confirmed or suspected misidentified DNA barcodes on GenBank/BOLD (See Table S1).

**Table 1:** Conspecific divergences in DNA barcodes associated with different subspecies designations.

Subspecies (n^1^)	Note
*Allotinus leogoron leogoron* (1)	4% from a GenBank conspecific from Sipora Island, Indonesia. An image but no subspecies name was provided by the authors [31]. Two subspecies could be present on this island: *A. l. leogoron* found in Peninsula Malaysia or *A. l. batuensis* [54] as found in Batu Islands, Indonesia [34].
*Appias paulina distanti* (1)	>2.2% from “Early-Release” and a GenBank conspecific from Australia, most likely *A. p. ega* [55]. *A. p. ega* has been treated as a distinct species [34].
*Ariadne ariadne ariadne* (3)	Clustered closely with a conspecific from Southern Thailand [5] but >3.7% from conspecifics from India ( [39]; most likely *A. a. indica* [34]) and (presumably [56]) Japan (unknown subspecies). *A. a. indica* has been treated as a distinct species [34].
*Danaus genutia intermedius* (3)	>2% from a “Private” conspecific from Australia, most likely *D. g. alexis* [34]). *D. g. alexis* has been treated as a distinct species [34].
*Dichorragia nesimachus* *deiokes* (1)	Matched closely (98.2%) with *D. n. nesiotes* from Japan but 3.7% from a conspecific from Leyte, Philippines, probably *D. n. peisistratus* [34], and 1.8% from a “Private” conspecific from Taiwan, most likely *D. n. formosanus* [34].
*Dophla evelina compta* (1)	100% similarity with a conspecific from Thailand but >2.5% from two other BINs each housing a single conspecific. The most similar from Java, Indonesia [31], most likely *D. e. sikani* (previously regarded as a distinct species [34]). The more distant from Western Ghats, India [39], most likely *D. e. derma* [34].
*Drupadia theda thesmia* (2)	>3.7% from “Early-Release” *D. theda* from West Kalimantan, Indonesia, most likely *D. t. vanica* [34]. Also 1.9% distant from a conspecific from Southern Thailand [5], which based on the image on BOLD may be *D. t. renonga*.
*Eooxylides tharis distanti* (1)	<2% from conspecifics from Thailand but >2.3% from “Early-Release” conspecifics from West Sumatra, Indonesia, most likely *E. t. tharis* [34].
*Graphium agamemnon* *agamemnon* (1)	2.0% from “Early-Release” conspecifics from Papua New Guinea, most likely *G. a. ligatus* [34].
*Graphium aristeus hermocrates* (1)	4.3% from “Early-Release” conspecifics from Papua New Guinea and Australia. Subspecies not provided but most likely *G. a. parnatus*, formerly treated as a distinct species [34].
*Graphium sarpedon luctatius* (2)	Close similarity with conspecifics from Thailand, Taiwan, China, and *G. s. nipponus* from South Korea [57] but relatively distant (3%) from conspecifics from Australia and Papua New Guinea, most likely *G. s. choredon* [34].
*Junonia hedonia hedonia* (1)	2.4% from *J. hedonia* from Australia and Papua New Guinea, most likely *J. h. zelima* [34].
*Lamproptera meges* *virescens* (3)	>2.2% from a conspecific from Yunnan, China. In Yunnan, the *L. meges* are a different subspecies which previously had been treated as a distinct species, *L. amplifascia* [34].
*Lethe confusa enima* (1)	Between 1–3% from *L. confusa* from Yunnan, China. Yunnan is the type locality of *L. c. confusa* [34].
*Lexias pardalis dirteana* (1)	2.8% from *L. pardalis* from Hainan and Vietnam, most likely *L. p. elenor* [34].
*Mycalesis anapita anapita* (1)	2.3% from *M. anapita* from Borneo, most likely *M. a. fucentia* [34].
*Mycalesis jardnardana* *sagittergera* (1)	5.8% from *M. jardnardana* from Sulawesi, Indonesia, most likely *M. j. opaculus* [34]. Morphological identification of *Mycalesis* butterflies is notoriously difficult so caution must be observed with the current taxonomic determinations of all *Mycalesis* DNA barcodes.
*Orsotriaena medus cinerea* (1)	100% similarity with *O. medus* from Bangladesh, Hainan and Southern Thailand, but >3% from *O. medus* from Papua New Guinea. The nominal subspecies is found in Papua New Guinea but also in the Indian subcontinent, south China and Thailand [34].
*Papilio helenus helenus* (3)	3.8% from a “Private” conspecific from Taiwan, most likely *P. h. fortunius* [34], 2.6% from *P. h. enganius* from Indonesia, and 1.4% from *P. helenus* from Japan, subspecies undetermined.
*Papilio nephelus sunatus* (3)	>2.3% from *P. n. chaon* from Thailand, Taiwan and China. The relatively large genetic distance between these two subspecies was also reported by Tsao and Yeh [58] although they regarded the Malaysian GenBank sequence (AY457579) as *P. n. chaon* (see the image [32]), which we regard as *P. n. sunatus*. These two taxa have previously been treated as distinct species [4]. Both subspecies are reported from Peninsula Malaysia, but where the third native taxon, *P. n. annulus,* an “intermediate” race [4], fits into this picture remains to be seen.
*Phalanta alcippe alcesta* (1)	>3% from the only conspecific on BOLD a DNA barcode from Taiwan of undetermined subspecies.
*Phalanta phalanta phalanta* (1)	Close similarity with conspecifics from India and Pakistan but 4.5% from conspecifics from Africa, probably *P. p. aethiopica* [34], and 2.5% from conspecifics from Australia, probably *P. p. araca* [34].
*Prothoe franck uniformis* (1)	>4% from the only conspecific on BOLD a DNA barcode of the nominal subspecies from Java, Indonesia.
*Spindasis lohita senama* (1)	4.6% from the only conspecific on BOLD a “Private” DNA barcode from Taiwan, most likely *S. l. formosana* [34].
*Thaumantis klugius lucipor* (2)	3.2% from *T. k. klugius* from Sabah, Malaysia (Borneo) [59].
*Vagrans egista* *macromalayana* (1)	8.6% from *V. egista* from Papua New Guinea which clustered with conspecifics from Australia (subspecies not given but most likely *V. e. propinqua*). *V. e. macromalayana* and *V. e. propinqua* have both been treated as distinct species [34] and wing patterns within this group are highly variable (see examples on BOLD [25]), with *V. e. macromalayana* having a dark brownish-black border along the costal edge of the forewing [4].
*Zizula hylax* (1)	Close similarity with *Z. h. hylax* from Thailand, Madagaskar and Africa but 3.7% from *Z. h. attenuata* from Australia, a subspecies previously treated as a species [34]. Only *Z. h. pygmaea* is in Corbet and Pendlebury [4] but *Z. h. hylax* has also been reported from Peninsula Malaysia [34].

1 DNA barcodes from UMKL.

### DNA Barcode Reference Libraries and Subspecies

In this study we present a preliminary DNA barcode library for a major component of the true butterfly species of Peninsula Malaysia. The majority of species and subspecies sampled possessed unique DNA barcodes. Although there is no fixed threshold of genetic distances clearly differentiating conspecific from interspecific distances, BOLD identification trees generally show a discernible pattern of low conspecific distances compared to interspecific distances, so can enable effective assignment of unknown DNA barcodes to species, especially when examined in conjunction with the BIN system.

Unlike assignment to a species, assignment of an unknown DNA barcode to a subspecies using a BOLD identification tree would not be easily accomplished. The genetic distances between most conspecific subspecies are small and indistinguishable from distances between members of the same subspecies. Although the majority of subspecies with multiple representatives formed exclusive clusters on Neighbor-joining trees in our analyses, forming an exclusive cluster cannot logically guide taxonomic assignments in the absence of other discernible patterns - exclusive clusters are present at, and between, all taxonomic levels on a tree [19].

Those subspecies that show ‘large’ inter-taxa distances probably warrant full species status. Conversely, there are undoubtedly cases where subspecies names are applied to groups that probably do not warrant taxonomic recognition [7], [12]. For example, considering the similarity of *Loxura atymnus* and *L. cassiopea* the necessity for finer taxonomic divisions [53] is dubious. Subspecies designations as shorthand for geographically and morphologically differentiated groups provide a useful heuristic for assessing how such groups correlate with clustering patterns of DNA barcodes, especially as the number of DNA barcodes per species in reference libraries increases. Considering this, we feel there is significant value in attaching subspecies names to records in DNA barcode databases. A beneficial addition to BOLD would be the facility to allow data contributors to specify subspecies names while still recognising that members of different subspecies are conspecific for the purpose of progress statistics and other analytical tools.

## Supporting Information

Table S1 Details of the specimens codes, including GenBank accessions, used in this study and results of screening of DNA barcodes.
(XLSX)

BIN S1 Tree for Barcode Index Number - BIN6082[BOLD:AAA6082]
(PDF)

BIN S2 Tree for Barcode Index Number - BIN511656[BOLD:ABZ1656]
(PDF)

BIN S3 Tree for Barcode Index Number - BIN108780[BOLD:AAK8780]
(PDF)

BIN S4 Tree for Barcode Index Number - BIN21308[BOLD:AAC1308]
(PDF)

Euploea Tree S1 Result of BOLD tree based identification of a UMKL DNA barcode for *Euploea modesta modesta*
(PDF)

Subspecies Trees S1 Trees resulting from a BOLD tree based identification of UMKL DNA barcodes
(PDF)
