# Supplementary material for: Building a DNA Barcode Reference Library for the True Butterflies (Lepidoptera) of Peninsula Malaysia: What about the Subspecies?
Source: PLoS One. 2013 Nov 25;8(11):e79969. doi: 10.1371/journal.pone.0079969 (PMC3839974; doi:10.1371/journal.pone.0079969)
Supplement: BIN S1 — Tree for Barcode Index Number - BIN6082[BOLD:AAA6082] (PDF) [file pone.0079969.s002.pdf]

BOLD TaxonID Tree

Title	:	Tree for Barcode Index Number - BIN6082[BOLD:AAA6082]
Date	:	12-June-2013
Data Type	:	Nucleotide
Distance Model	:	Kimura 2 Parameter
Codon Positions	:	1st, 2nd, 3rd
Labels	:	Country & Province, ProcessID, BIN name

Sequence Count	:	51
Species count	:	7
Genus count	:	1
Family count	:	1
Unidentified	:	2

BIN Count	:	2

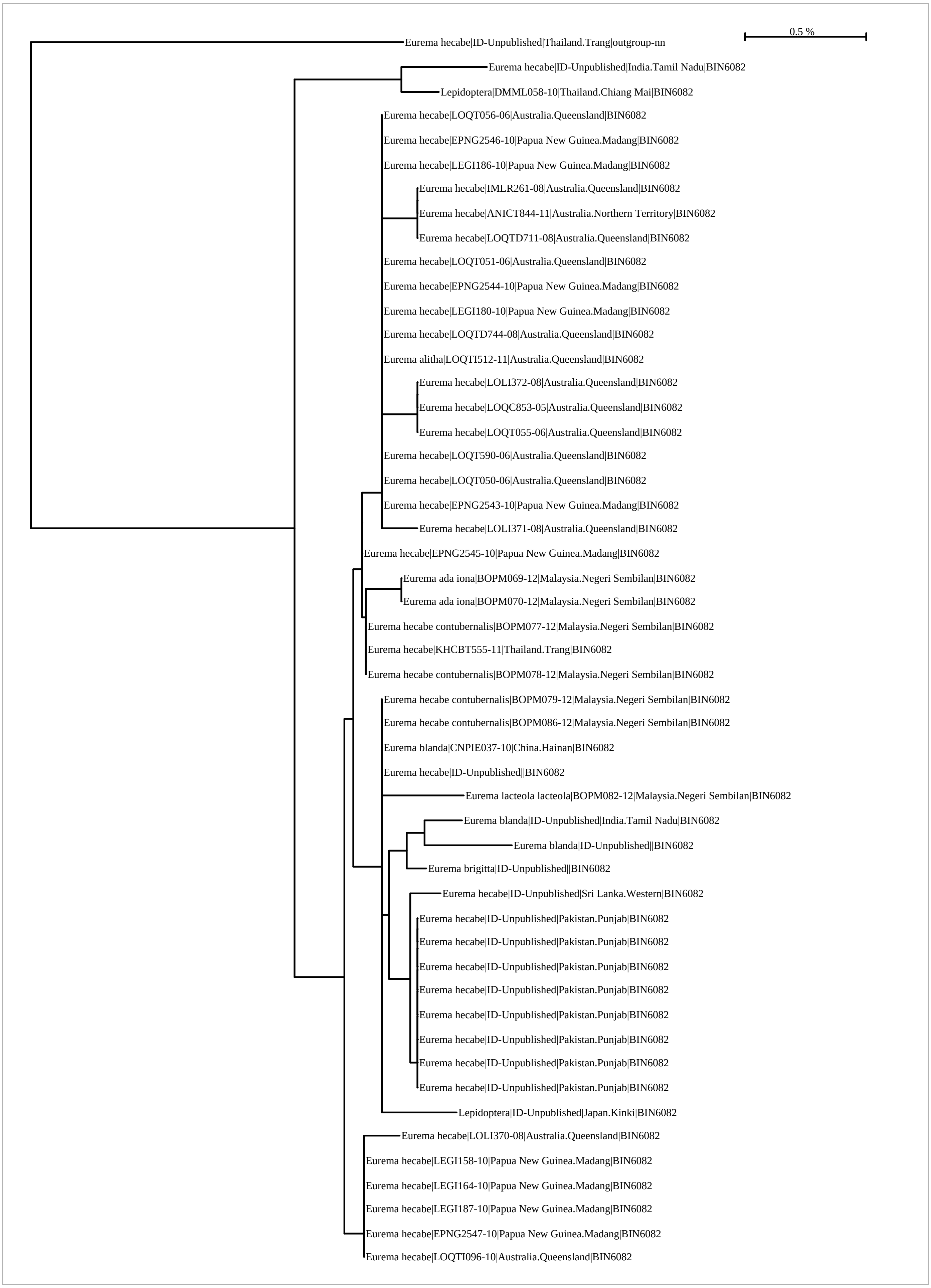
0.5 %
Eurema hecabe|ID-Unpublished|Thailand.Trang|outgroup-nn
Eurema hecabe|ID-Unpublished|India.Tamil Nadu|BIN6082
Lepidoptera|DMML058-10|Thailand.Chiang Mai|BIN6082
Eurema hecabe|LOQT056-06|Australia.Queensland|BIN6082
Eurema hecabe|EPNG2546-10|Papua New Guinea.Madang|BIN6082
Eurema hecabe|LEGI186-10|Papua New Guinea.Madang|BIN6082
Eurema hecabe|IMLR261-08|Australia.Queensland|BIN6082
Eurema hecabe|ANICT844-11|Australia.Northern Territory|BIN6082
Eurema hecabe|LOQTD711-08|Australia.Queensland|BIN6082
Eurema hecabe|LOQT051-06|Australia.Queensland|BIN6082
Eurema hecabe|EPNG2544-10|Papua New Guinea.Madang|BIN6082
Eurema hecabe|LEGI180-10|Papua New Guinea.Madang|BIN6082
Eurema hecabe|LOQTD744-08|Australia.Queensland|BIN6082
Eurema alitha|LOQTI512-11|Australia.Queensland|BIN6082
Eurema hecabe|LOLI372-08|Australia.Queensland|BIN6082
Eurema hecabe|LOQC853-05|Australia.Queensland|BIN6082
Eurema hecabe|LOQT055-06|Australia.Queensland|BIN6082
Eurema hecabe|LOQT590-06|Australia.Queensland|BIN6082
Eurema hecabe|LOQT050-06|Australia.Queensland|BIN6082
Eurema hecabe|EPNG2543-10|Papua New Guinea.Madang|BIN6082
Eurema hecabe|LOLI371-08|Australia.Queensland|BIN6082
Eurema hecabe|EPNG2545-10|Papua New Guinea.Madang|BIN6082
Eurema ada iona|BOPM069-12|Malaysia.Negeri Sembilan|BIN6082
Eurema ada iona|BOPM070-12|Malaysia.Negeri Sembilan|BIN6082
Eurema hecabe contubernalis|BOPM077-12|Malaysia.Negeri Sembilan|BIN6082
Eurema hecabe|KHCBT555-11|Thailand.Trang|BIN6082
Eurema hecabe contubernalis|BOPM078-12|Malaysia.Negeri Sembilan|BIN6082
Eurema hecabe contubernalis|BOPM079-12|Malaysia.Negeri Sembilan|BIN6082
Eurema hecabe contubernalis|BOPM086-12|Malaysia.Negeri Sembilan|BIN6082
Eurema blanda|CNPIE037-10|China.Hainan|BIN6082
Eurema hecabe|ID-Unpublished||BIN6082
Eurema lacteola lacteola|BOPM082-12|Malaysia.Negeri Sembilan|BIN6082
Eurema blanda|ID-Unpublished|India.Tamil Nadu|BIN6082
Eurema blanda|ID-Unpublished||BIN6082
Eurema brigitta|ID-Unpublished||BIN6082
Eurema hecabe|ID-Unpublished|Sri Lanka.Western|BIN6082
Eurema hecabe|ID-Unpublished|Pakistan.Punjab|BIN6082
Eurema hecabe|ID-Unpublished|Pakistan.Punjab|BIN6082
Eurema hecabe|ID-Unpublished|Pakistan.Punjab|BIN6082
Eurema hecabe|ID-Unpublished|Pakistan.Punjab|BIN6082
Eurema hecabe|ID-Unpublished|Pakistan.Punjab|BIN6082
Eurema hecabe|ID-Unpublished|Pakistan.Punjab|BIN6082
Eurema hecabe|ID-Unpublished|Pakistan.Punjab|BIN6082
Eurema hecabe|ID-Unpublished|Pakistan.Punjab|BIN6082
Lepidoptera|ID-Unpublished|Japan.Kinki|BIN6082
Eurema hecabe|LOLI370-08|Australia.Queensland|BIN6082
Eurema hecabe|LEGI158-10|Papua New Guinea.Madang|BIN6082
Eurema hecabe|LEGI164-10|Papua New Guinea.Madang|BIN6082
Eurema hecabe|LEGI187-10|Papua New Guinea.Madang|BIN6082
Eurema hecabe|EPNG2547-10|Papua New Guinea.Madang|BIN6082
Eurema hecabe|LOQTI096-10|Australia.Queensland|BIN6082
