# Supplementary material for: Building a DNA Barcode Reference Library for the True Butterflies (Lepidoptera) of Peninsula Malaysia: What about the Subspecies?
Source: PLoS One. 2013 Nov 25;8(11):e79969. doi: 10.1371/journal.pone.0079969 (PMC3839974; doi:10.1371/journal.pone.0079969)
Supplement: BIN S2 — Tree for Barcode Index Number - BIN511656[BOLD:ABZ1656] (PDF) [file pone.0079969.s003.pdf]

BOLD TaxonID Tree

Title	:	Tree for Barcode Index Number - BIN511656[BOLD:ABZ1656]
Date	:	12-June-2013
Data Type	:	Nucleotide
Distance Model	:	Kimura 2 Parameter
Codon Positions	:	1st, 2nd, 3rd
Labels	:	Country & Province, ProcessID, BIN name

Sequence Count	:	11
Species count	:	6
Genus count	:	1
Family count	:	1
Unidentified	:	0

BIN Count	:	2

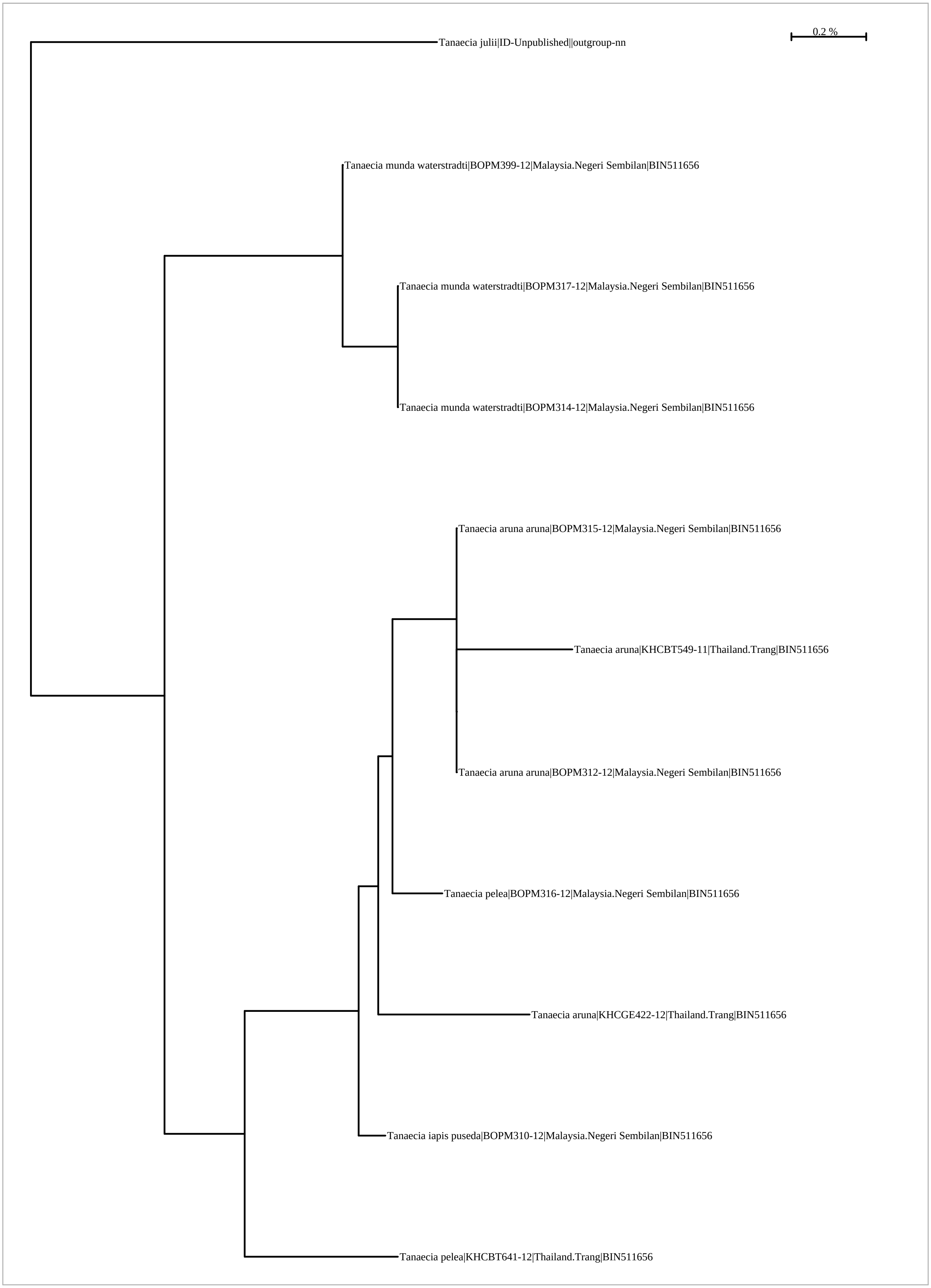
0.2 %
Tanaecia julii|ID-Unpublished||outgroup-nn
Tanaecia munda waterstradti|BOPM399-12|Malaysia.Negeri Sembilan|BIN511656
Tanaecia munda waterstradti|BOPM317-12|Malaysia.Negeri Sembilan|BIN511656
Tanaecia munda waterstradti|BOPM314-12|Malaysia.Negeri Sembilan|BIN511656
Tanaecia aruna aruna|BOPM315-12|Malaysia.Negeri Sembilan|BIN511656
Tanaecia aruna|KHCBT549-11|Thailand.Trang|BIN511656
Tanaecia aruna aruna|BOPM312-12|Malaysia.Negeri Sembilan|BIN511656
Tanaecia pelea|BOPM316-12|Malaysia.Negeri Sembilan|BIN511656
Tanaecia aruna|KHCGE422-12|Thailand.Trang|BIN511656
Tanaecia iapis puseda|BOPM310-12|Malaysia.Negeri Sembilan|BIN511656
Tanaecia pelea|KHCBT641-12|Thailand.Trang|BIN511656
