# Supplementary material for: Building a DNA Barcode Reference Library for the True Butterflies (Lepidoptera) of Peninsula Malaysia: What about the Subspecies?
Source: PLoS One. 2013 Nov 25;8(11):e79969. doi: 10.1371/journal.pone.0079969 (PMC3839974; doi:10.1371/journal.pone.0079969)

BOLD TaxonID Tree

Title	:	Tree for Barcode Index Number - BIN108780[BOLD:AAK8780]
Date	:	28-July-2013
Data Type	:	Nucleotide
Distance Model	:	Kimura 2 Parameter
Codon Positions	:	1st, 2nd, 3rd
Labels	:	Country & Province, ProcessID, BIN name

Sequence Count	:	20
Species count	:	6
Genus count	:	1
Family count	:	1
Unidentified	:	1

BIN Count	:	2

Tree_for_Barcode_Index_Number_-_BIN108780[BOLD:AAK8780] Sun Jul 28 05:25:28 2013 Page 1 of 1

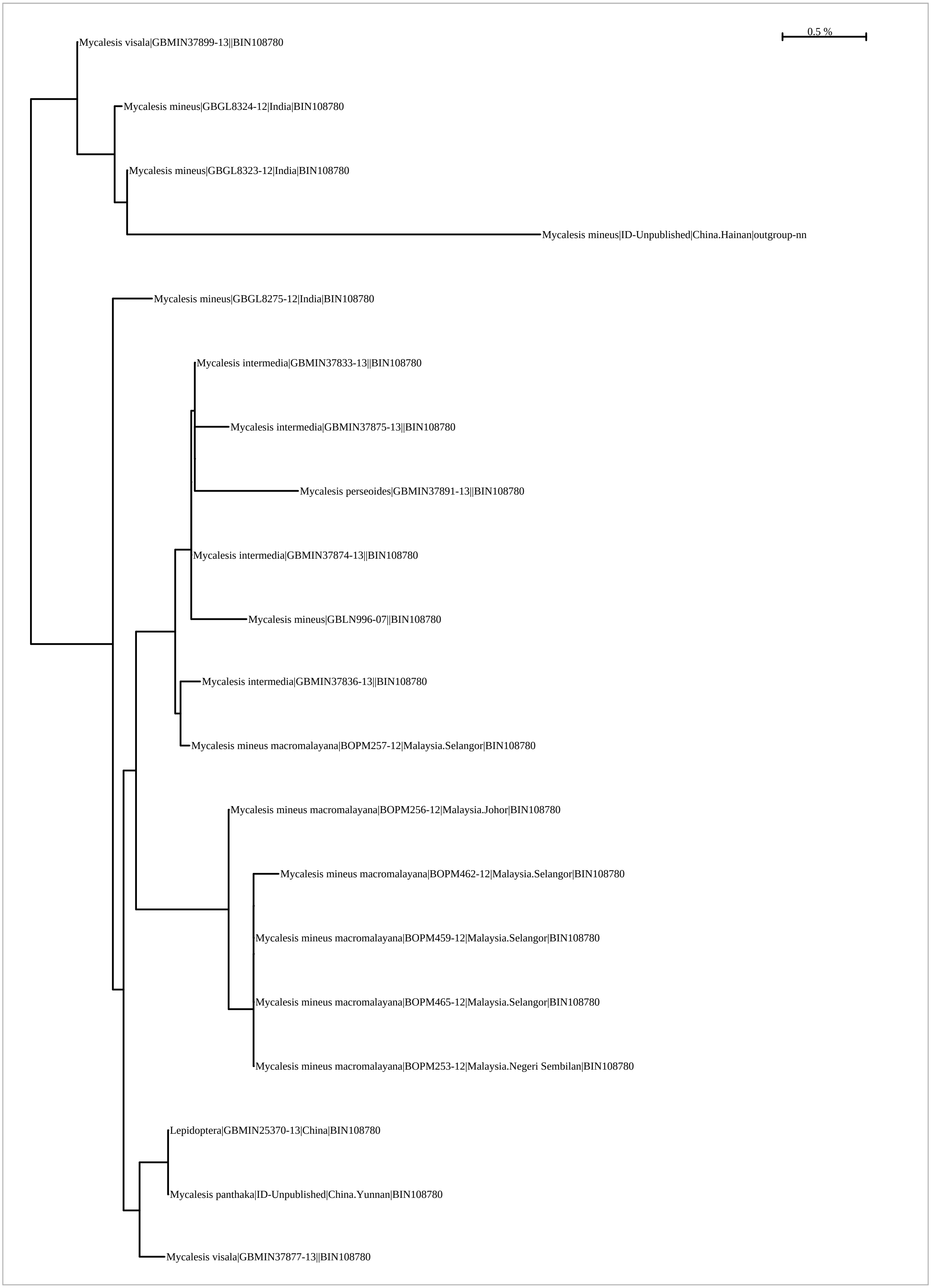

Supplement: BIN S3 — Tree for Barcode Index Number - BIN108780[BOLD:AAK8780] (PDF) [file pone.0079969.s004.pdf]
