# Supplementary material for: Building a DNA Barcode Reference Library for the True Butterflies (Lepidoptera) of Peninsula Malaysia: What about the Subspecies?
Source: PLoS One. 2013 Nov 25;8(11):e79969. doi: 10.1371/journal.pone.0079969 (PMC3839974; doi:10.1371/journal.pone.0079969)
Supplement: BIN S4 — Tree for Barcode Index Number - BIN21308[BOLD:AAC1308] (PDF) [file pone.0079969.s005.pdf]

BOLD TaxonID Tree

Title	:	Tree for Barcode Index Number - BIN21308[BOLD:AAC1308]
Date	:	30-July-2013
Data Type	:	Nucleotide
Distance Model	:	Kimura 2 Parameter
Codon Positions	:	1st, 2nd, 3rd
Labels	:	Country & Province, ProcessID, BIN name
Sequence Count	:	15
Species count	:	5
Genus count	:	2
Family count	:	1
Unidentified	:	0
BIN Count	:	2

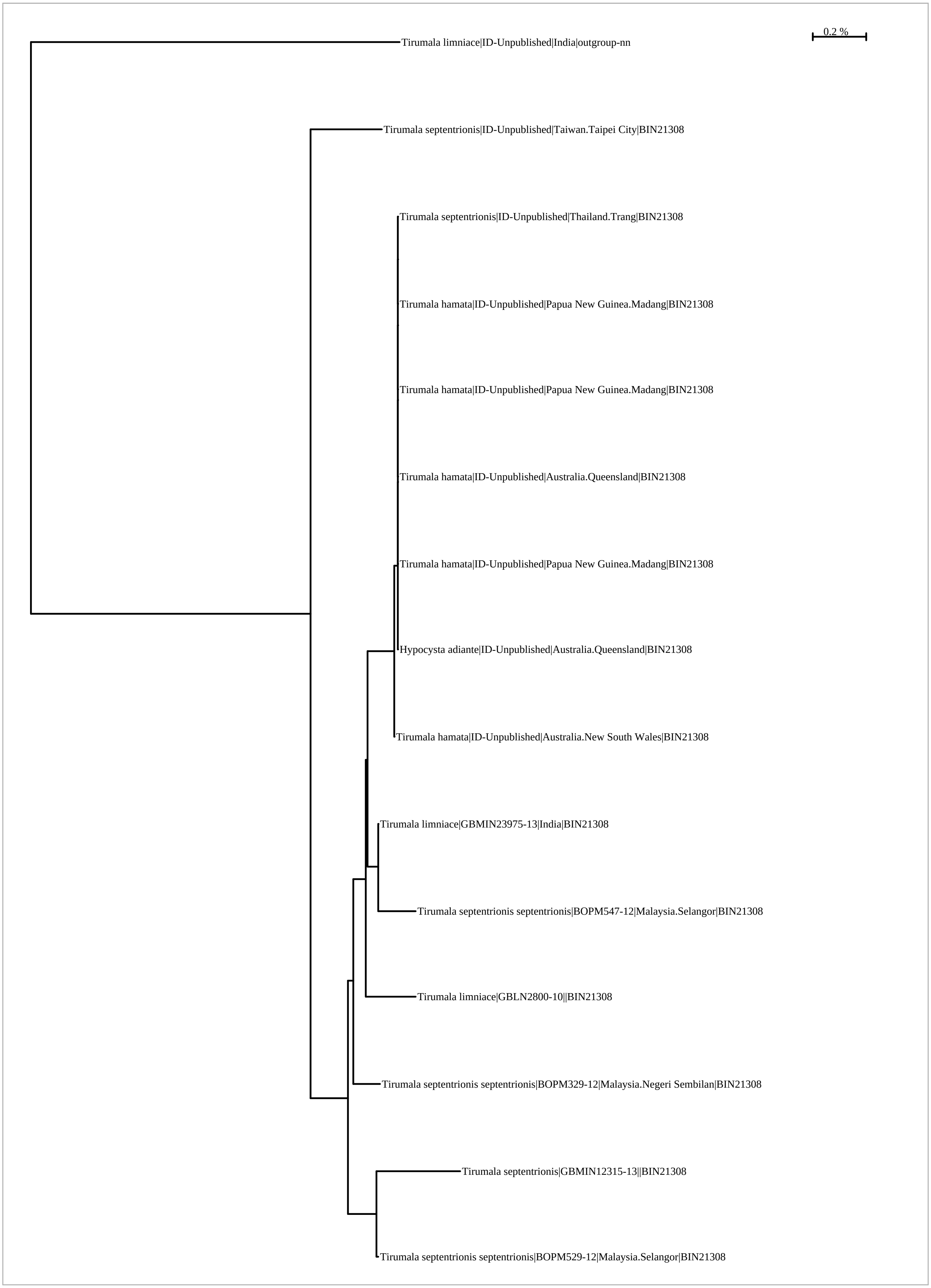
0.2 %
Tirumala limniace|ID-Unpublished|India|outgroup-nn
Tirumala septentrionis|ID-Unpublished|Taiwan.Taipei City|BIN21308
Tirumala septentrionis|ID-Unpublished|Thailand.Trang|BIN21308
Tirumala hamata|ID-Unpublished|Papua New Guinea.Madang|BIN21308
Tirumala hamata|ID-Unpublished|Papua New Guinea.Madang|BIN21308
Tirumala hamata|ID-Unpublished|Australia.Queensland|BIN21308
Tirumala hamata|ID-Unpublished|Papua New Guinea.Madang|BIN21308
Hypocysta adiante|ID-Unpublished|Australia.Queensland|BIN21308
Tirumala hamata|ID-Unpublished|Australia.New South Wales|BIN21308
Tirumala limniace|GBMIN23975-13|India|BIN21308
Tirumala septentrionis septentrionis|BOPM547-12|Malaysia.Selangor|BIN21308
Tirumala limniace|GBLN2800-10||BIN21308
Tirumala septentrionis septentrionis|BOPM329-12|Malaysia.Negeri Sembilan|BIN21308
Tirumala septentrionis|GBMIN12315-13||BIN21308
Tirumala septentrionis septentrionis|BOPM529-12|Malaysia.Selangor|BIN21308
