# Supplementary material for: Building a DNA Barcode Reference Library for the True Butterflies (Lepidoptera) of Peninsula Malaysia: What about the Subspecies?
Source: PLoS One. 2013 Nov 25;8(11):e79969. doi: 10.1371/journal.pone.0079969 (PMC3839974; doi:10.1371/journal.pone.0079969)
Supplement: Euploea Tree S1 — Result of BOLD tree based identification of a UMKL DNA barcode for Euploea modesta modesta (PDF) [file pone.0079969.s006.pdf]

BOLD TaxonID Tree

Title	:	COI FULL DATABASE includes records without species designati...
Date	:	31-July-2013
Data Type	:	Nucleotide
Distance Model	:	Kimura 2 Parameter
Marker	:	COI-5P
Codon Positions	:	1st, 2nd, 3rd
Labels	:	Extra Info, Country & Province, Family
Filters	:	Length > 200
Attachment	:	Photographs & Spreadsheet

Sequence Count	:	100
Species count	:	22
Genus count	:	1
Family count	:	1
Unidentified	:	4

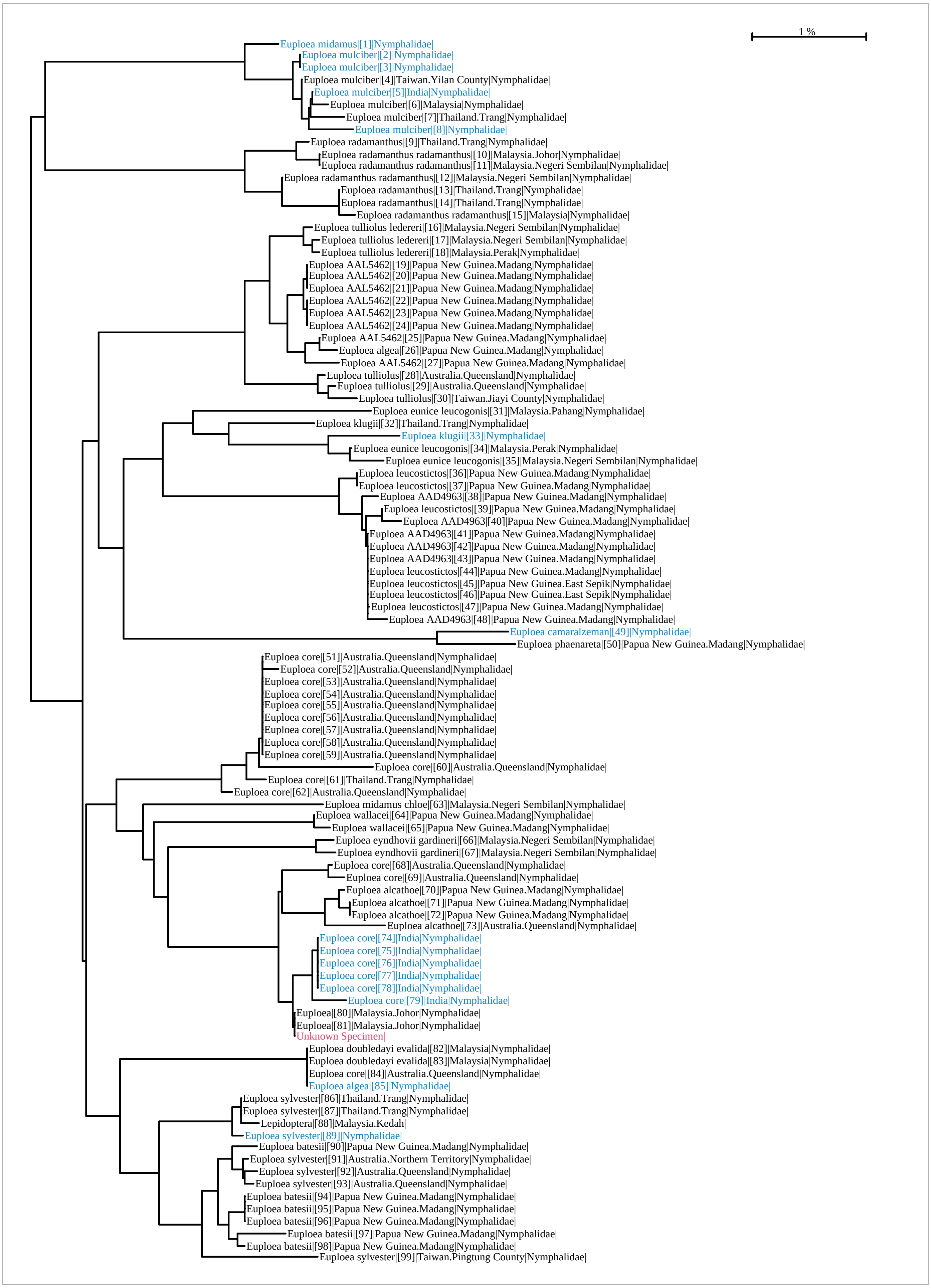
1 %
Euploea midamus|[1]|Nymphalidae|
Euploea mulciber|[2]|Nymphalidae|
Euploea mulciber|[3]|Nymphalidae|
Euploea mulciber|[4]|Taiwan.Yilan County|Nymphalidae|
Euploea mulciber|[5]|India|Nymphalidae|
Euploea mulciber|[6]|Malaysia|Nymphalidae|
Euploea mulciber|[7]|Thailand.Trang|Nymphalidae|
Euploea mulciber|[8]|Nymphalidae|
Euploea radamanthus|[9]|Thailand.Trang|Nymphalidae|
Euploea radamanthus radamanthus|[10]|Malaysia.Johor|Nymphalidae|
Euploea radamanthus radamanthus|[11]|Malaysia.Negeri Sembilan|Nymphalidae|
Euploea radamanthus radamanthus|[12]|Malaysia.Negeri Sembilan|Nymphalidae|
Euploea radamanthus|[13]|Thailand.Trang|Nymphalidae|
Euploea radamanthus|[14]|Thailand.Trang|Nymphalidae|
Euploea radamanthus radamanthus|[15]|Malaysia|Nymphalidae|
Euploea tulliolus ledereri|[16]|Malaysia.Negeri Sembilan|Nymphalidae|
Euploea tulliolus ledereri|[17]|Malaysia.Negeri Sembilan|Nymphalidae|
Euploea tulliolus ledereri|[18]|Malaysia.Perak|Nymphalidae|
Euploea AAL5462|[19]|Papua New Guinea.Madang|Nymphalidae|
Euploea AAL5462|[20]|Papua New Guinea.Madang|Nymphalidae|
Euploea AAL5462|[21]|Papua New Guinea.Madang|Nymphalidae|
Euploea AAL5462|[22]|Papua New Guinea.Madang|Nymphalidae|
Euploea AAL5462|[23]|Papua New Guinea.Madang|Nymphalidae|
Euploea AAL5462|[24]|Papua New Guinea.Madang|Nymphalidae|
Euploea AAL5462|[25]|Papua New Guinea.Madang|Nymphalidae|
Euploea algea|[26]|Papua New Guinea.Madang|Nymphalidae|
Euploea AAL5462|[27]|Papua New Guinea.Madang|Nymphalidae|
Euploea tulliolus|[28]|Australia.Queensland|Nymphalidae|
Euploea tulliolus|[29]|Australia.Queensland|Nymphalidae|
Euploea tulliolus|[30]|Taiwan.Jiayi County|Nymphalidae|
Euploea eunice leucogonis|[31]|Malaysia.Pahang|Nymphalidae|
Euploea klugii|[32]|Thailand.Trang|Nymphalidae|
Euploea klugii|[33]|Nymphalidae|
Euploea eunice leucogonis|[34]|Malaysia.Perak|Nymphalidae|
Euploea eunice leucogonis|[35]|Malaysia.Negeri Sembilan|Nymphalidae|
Euploea leucostictos|[36]|Papua New Guinea.Madang|Nymphalidae|
Euploea leucostictos|[37]|Papua New Guinea.Madang|Nymphalidae|
Euploea AAD4963|[38]|Papua New Guinea.Madang|Nymphalidae|
Euploea leucostictos|[39]|Papua New Guinea.Madang|Nymphalidae|
Euploea AAD4963|[40]|Papua New Guinea.Madang|Nymphalidae|
Euploea AAD4963|[41]|Papua New Guinea.Madang|Nymphalidae|
Euploea AAD4963|[42]|Papua New Guinea.Madang|Nymphalidae|
Euploea AAD4963|[43]|Papua New Guinea.Madang|Nymphalidae|
Euploea leucostictos|[44]|Papua New Guinea.Madang|Nymphalidae|
Euploea leucostictos|[45]|Papua New Guinea.East Sepik|Nymphalidae|
Euploea leucostictos|[46]|Papua New Guinea.East Sepik|Nymphalidae|
Euploea leucostictos|[47]|Papua New Guinea.Madang|Nymphalidae|
Euploea AAD4963|[48]|Papua New Guinea.Madang|Nymphalidae|
Euploea camaralzeman|[49]|Nymphalidae|
Euploea phaenareta|[50]|Papua New Guinea.Madang|Nymphalidae|
Euploea core|[51]|Australia.Queensland|Nymphalidae|
Euploea core|[52]|Australia.Queensland|Nymphalidae|
Euploea core|[53]|Australia.Queensland|Nymphalidae|
Euploea core|[54]|Australia.Queensland|Nymphalidae|
Euploea core|[55]|Australia.Queensland|Nymphalidae|
Euploea core|[56]|Australia.Queensland|Nymphalidae|
Euploea core|[57]|Australia.Queensland|Nymphalidae|
Euploea core|[58]|Australia.Queensland|Nymphalidae|
Euploea core|[59]|Australia.Queensland|Nymphalidae|
Euploea core|[60]|Australia.Queensland|Nymphalidae|
Euploea core|[61]|Thailand.Trang|Nymphalidae|
Euploea core|[62]|Australia.Queensland|Nymphalidae|
Euploea midamus chloe|[63]|Malaysia.Negeri Sembilan|Nymphalidae|
Euploea wallacei|[64]|Papua New Guinea.Madang|Nymphalidae|
Euploea wallacei|[65]|Papua New Guinea.Madang|Nymphalidae|
Euploea eyndhovii gardineri|[66]|Malaysia.Negeri Sembilan|Nymphalidae|
Euploea eyndhovii gardineri|[67]|Malaysia.Negeri Sembilan|Nymphalidae|
Euploea core|[68]|Australia.Queensland|Nymphalidae|
Euploea core|[69]|Australia.Queensland|Nymphalidae|
Euploea alcathoe|[70]|Papua New Guinea.Madang|Nymphalidae|
Euploea alcathoe|[71]|Papua New Guinea.Madang|Nymphalidae|
Euploea alcathoe|[72]|Papua New Guinea.Madang|Nymphalidae|
Euploea alcathoe|[73]|Australia.Queensland|Nymphalidae|
Euploea core|[74]|India|Nymphalidae|
Euploea core|[75]|India|Nymphalidae|
Euploea core|[76]|India|Nymphalidae|
Euploea core|[77]|India|Nymphalidae|
Euploea core|[78]|India|Nymphalidae|
Euploea core|[79]|India|Nymphalidae|
Euploea|[80]|Malaysia.Johor|Nymphalidae|
Euploea|[81]|Malaysia.Johor|Nymphalidae|
Unknown Specimen|
Euploea doubledayi evalida|[82]|Malaysia|Nymphalidae|
Euploea doubledayi evalida|[83]|Malaysia|Nymphalidae|
Euploea core|[84]|Australia.Queensland|Nymphalidae|
Euploea algea|[85]|Nymphalidae|
Euploea sylvester|[86]|Thailand.Trang|Nymphalidae|
Euploea sylvester|[87]|Thailand.Trang|Nymphalidae|
Lepidoptera|[88]|Malaysia.Kedah|
Euploea sylvester|[89]|Nymphalidae|
Euploea batesii|[90]|Papua New Guinea.Madang|Nymphalidae|
Euploea sylvester|[91]|Australia.Northern Territory|Nymphalidae|
Euploea sylvester|[92]|Australia.Queensland|Nymphalidae|
Euploea sylvester|[93]|Australia.Queensland|Nymphalidae|
Euploea batesii|[94]|Papua New Guinea.Madang|Nymphalidae|
Euploea batesii|[95]|Papua New Guinea.Madang|Nymphalidae|
Euploea batesii|[96]|Papua New Guinea.Madang|Nymphalidae|
Euploea batesii|[97]|Papua New Guinea.Madang|Nymphalidae|
Euploea batesii|[98]|Papua New Guinea.Madang|Nymphalidae|
Euploea sylvester|[99]|Taiwan.Pingtung County|Nymphalidae|
