# Supplementary material for: Building a DNA Barcode Reference Library for the True Butterflies (Lepidoptera) of Peninsula Malaysia: What about the Subspecies?
Source: PLoS One. 2013 Nov 25;8(11):e79969. doi: 10.1371/journal.pone.0079969 (PMC3839974; doi:10.1371/journal.pone.0079969)

These trees come from a BOLD "Tree Based Identification" of a DNA barcode from the new Peninsula Malaysia library for species represented by more than one subspecies on BOLD (Refer to Table S1). Where more than one DNA barcode was available from the new Peninsula Malaysia library, the longest is shown as the Unknown in the trees, or in the case of equal length a randomly selected barcode.

The tree have been annotated with red lines separating different subspecies and species and red stars highlighting places where different subspecies share identical barcodes.

In some cases the "unknown" test sequence was short in length so had a spurious placement on the identification tree despite showing close similarity with conspecifics. In these cases the "Unknown Specimen" may not be shown on the subtree displayed here.

Tree 1 – 4 singleton unique; 3 multi identical; 1 multi unique

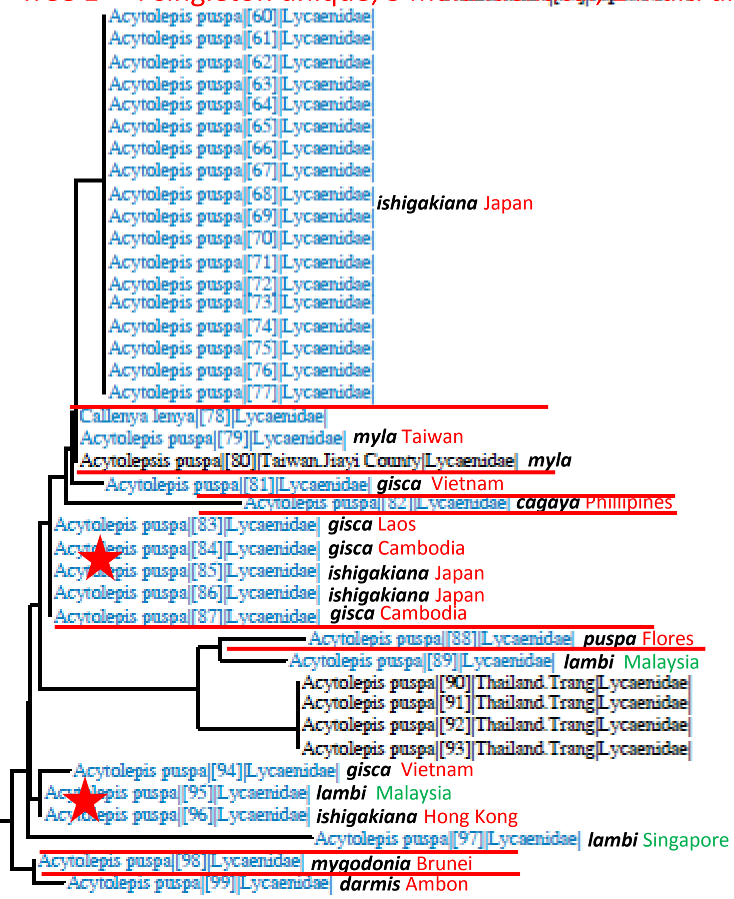

Tree 2 – 2 singleton unique; 1 multi unique

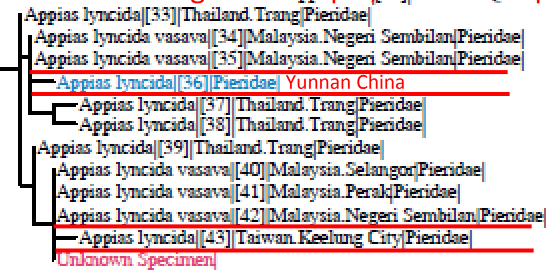

Tree 3 – 1 singleton unique; 1 multi exclusive

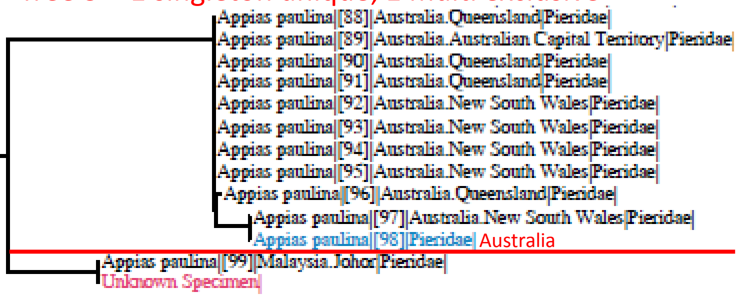

Tree 4 – 2 singleton unique; 1 multi exclusive

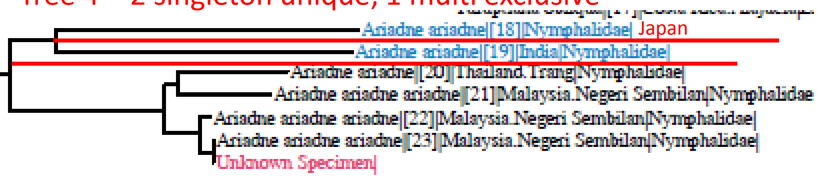

Tree 5 – 1 singleton unique; 1 multi exclusive

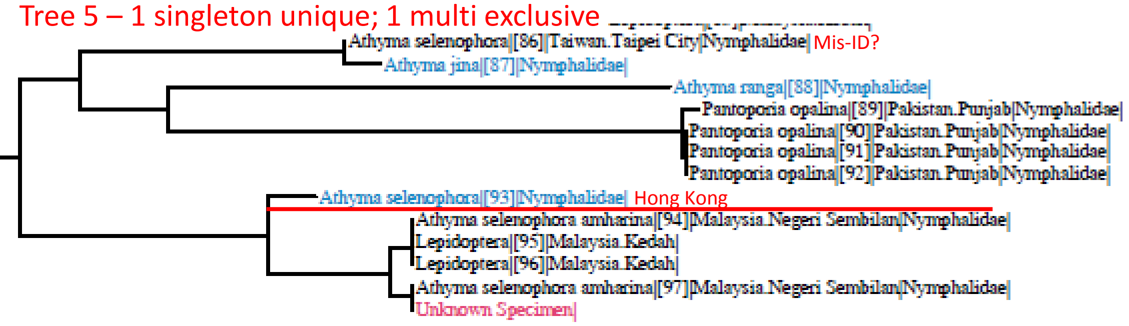

Tree 6 – 1 singleton unique; 1 multi exclusive

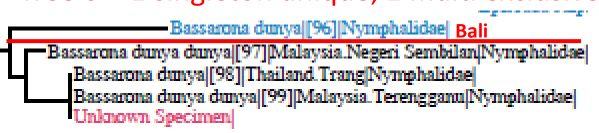

Tree 7 – 2 multi exclusive

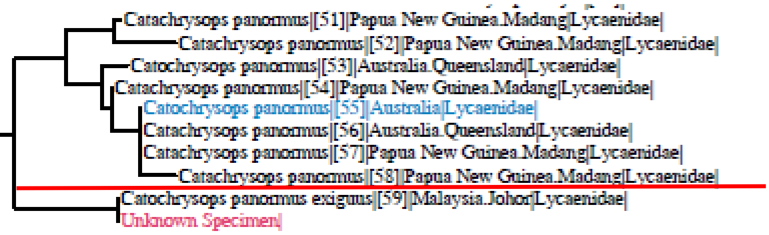

Tree 8 – 2 multi exclusive

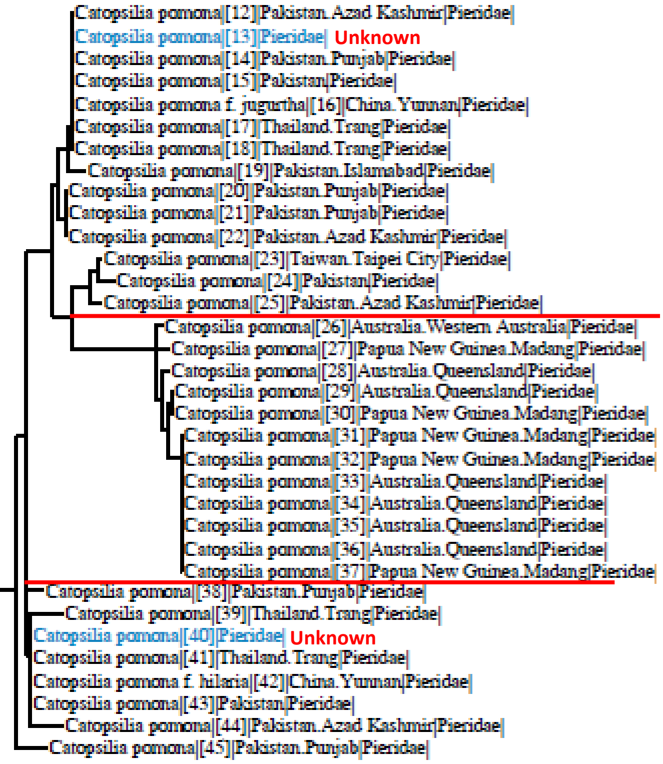

Tree 9 – 1 singleton identical; 1 multi identical

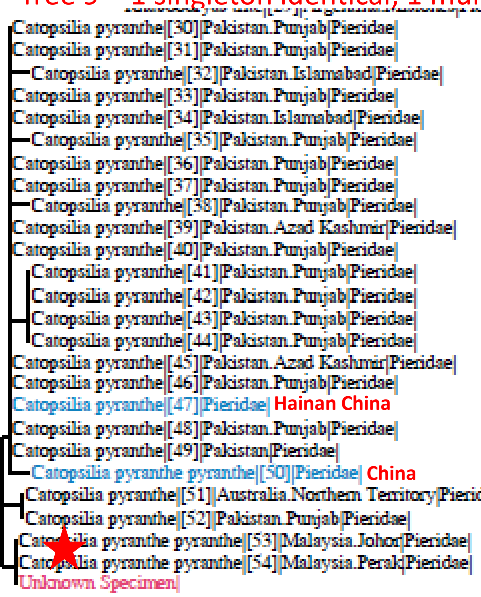

Tree 10 – 1 singleton unique; 1 multi exclusive

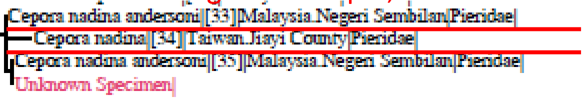

Tree 11 – 1 singleton unique; 1 multi unique; 1 multi exclusive

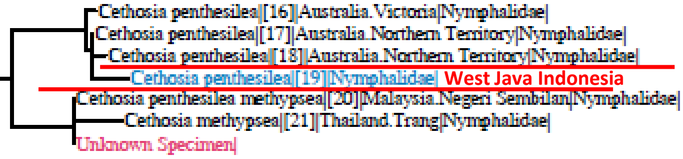

Tree 12 – 1 singleton identical; 1 multi identical

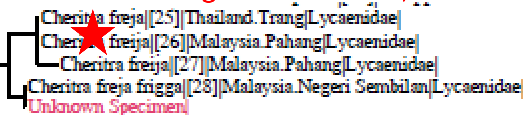

Tree 13 – 1 singleton identical; 1 multi identical

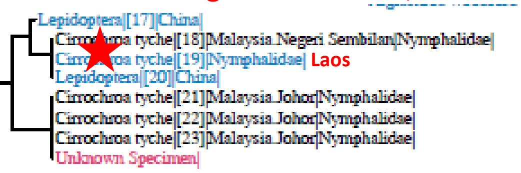

Tree 14 – 2 multi exclusive

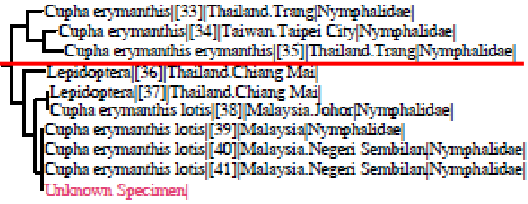

Tree 15 – 2 multi exclusive

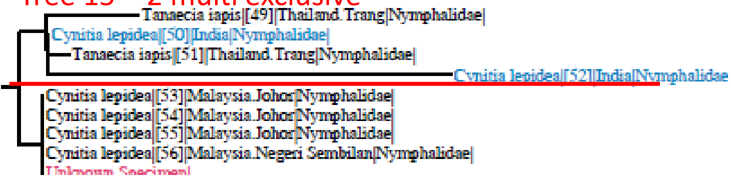

Tree 16 – 1 singleton unique; 1 multi exclusive

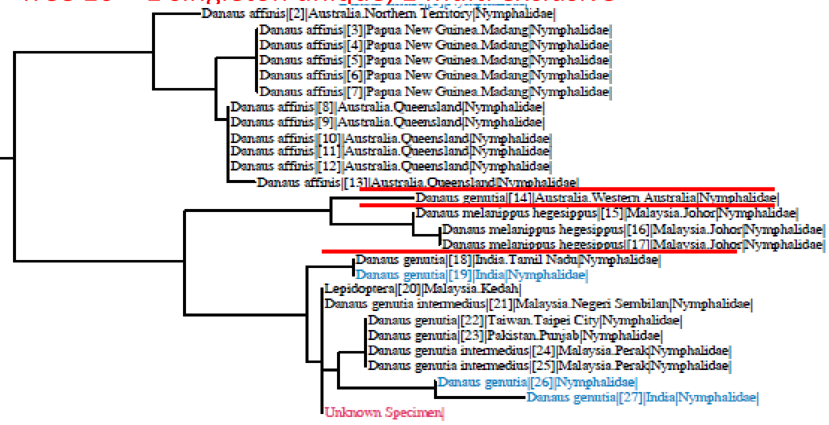

Tree 17 – 1 singleton unique; 2 multi unique

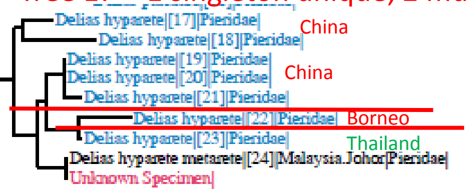

Tree 18 – 3 singleton unique

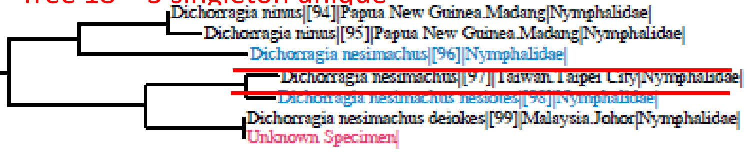

Tree 19 – 1 singleton identical; 1 multi identical

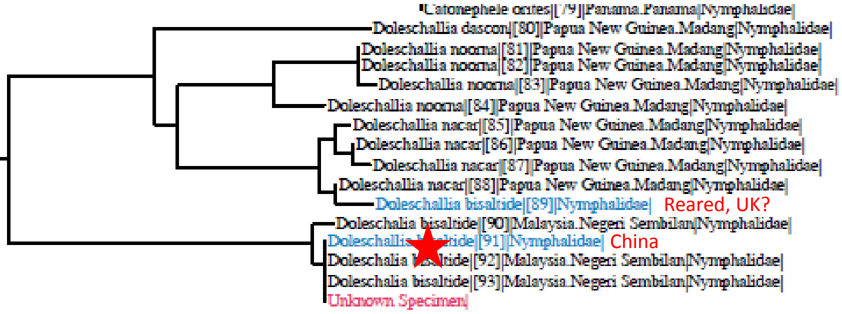

Tree 20 – 2 singleton unique; 1 multi exclusive

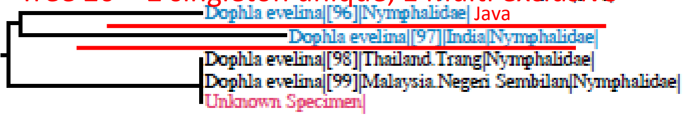

Tree 21 – 1 singleton unique; 2 multi exclusive

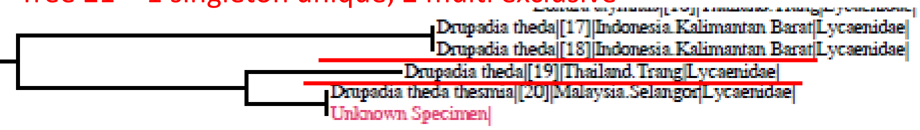

Tree 22 – 1 singleton unique; 1 multi unique; 2 multi exclusive

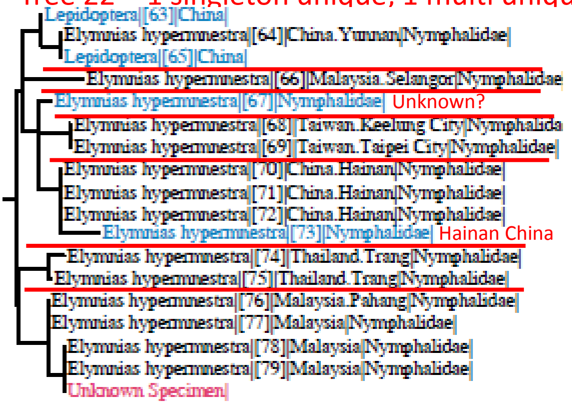

Tree 23 – 2 multi exclusive

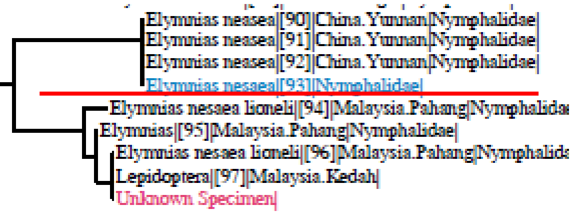

Tree 24 – 2 multi exculsive

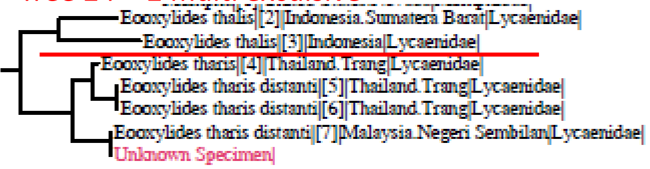

Tree 25 – 1 singleton unique; 1 multi exclusive

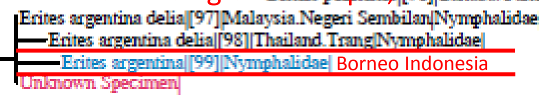

Tree 26 – I singleton unique; 1 multi exclusive

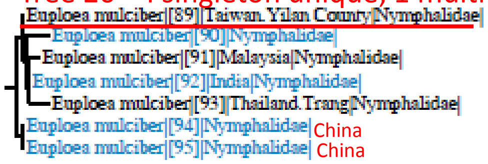

Tree 27 – 1 singleton unique; 1 multi unique; 1 multi exclusive

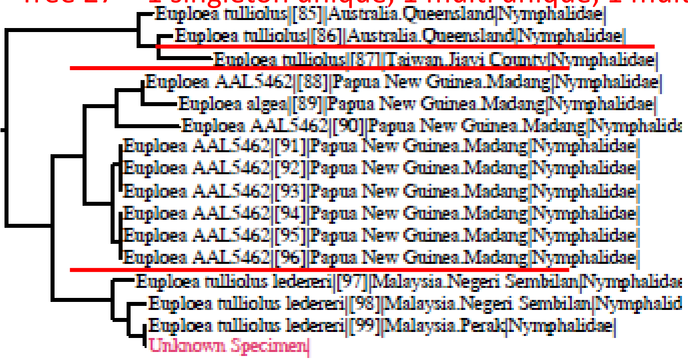

Tree 28 – 2 singleton identical; 1 multi unique; 2 multi exclusive

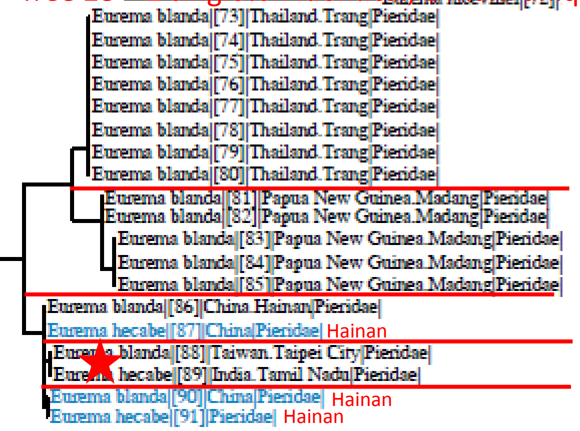

Tree 29 – 1 singleton unique; 1 multi exclusive

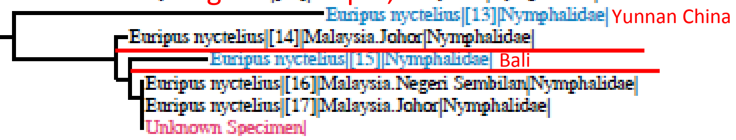

Tree 30 – 2 multi exclusive

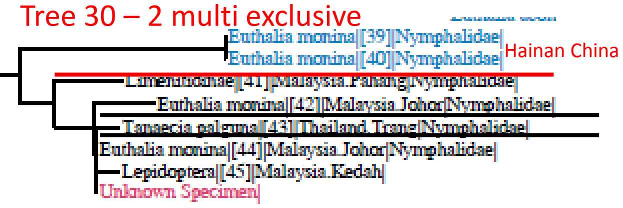

Tree 31 – 1 singleton unique; 1 multi exclusive

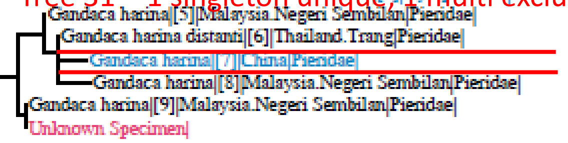

Tree 32 – 2 multi exclusive

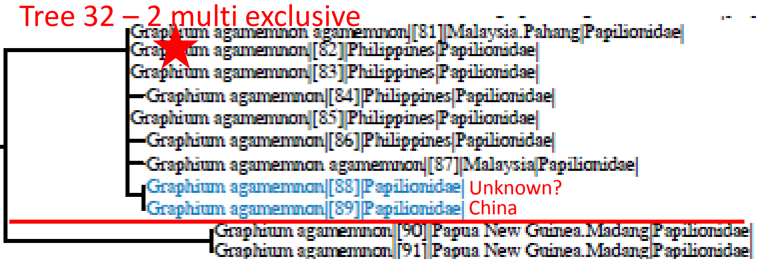

Tree 33 – 1 singleton unique; 1 multi exclusive

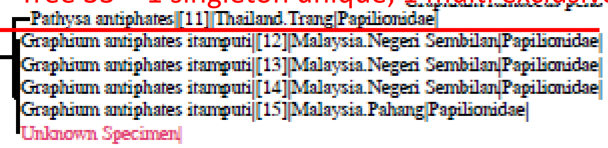

Tree 34 – 1 singleton unique; 1 multi exclusive

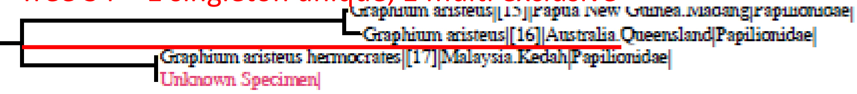

Tree 35 – 1 singleton unique; 1 multi exclusive

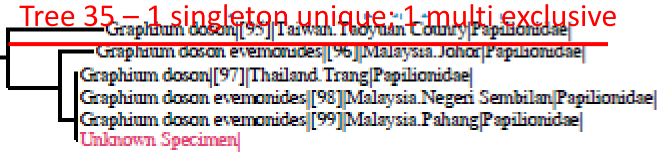

Tree 36 – 3 singletons unique; 2 multi exclusive

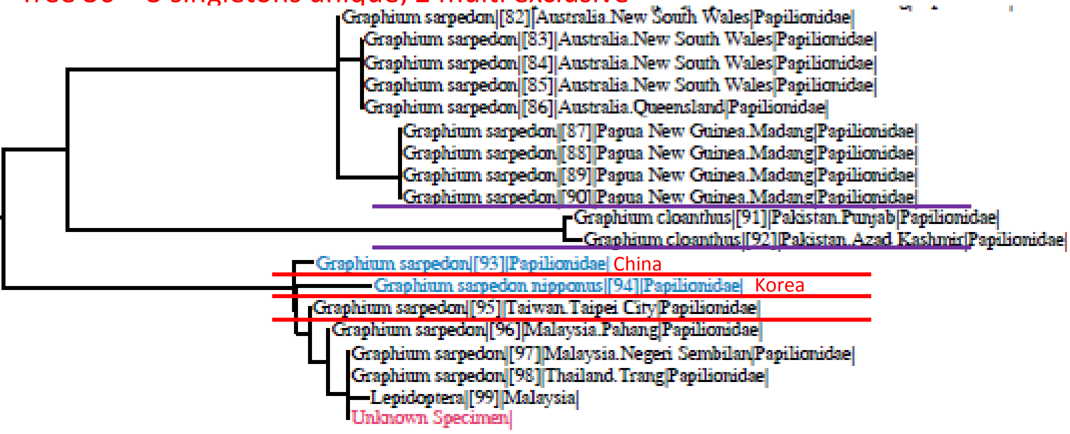

Tree 37 – 3 singletons unique; 1 multi unique

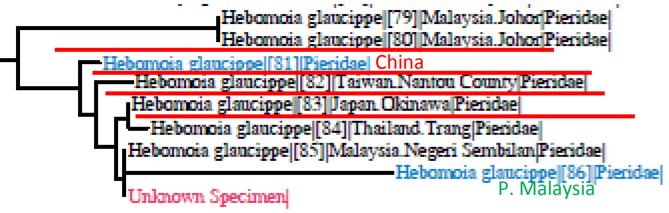

Tree 38 – 2 multi identical

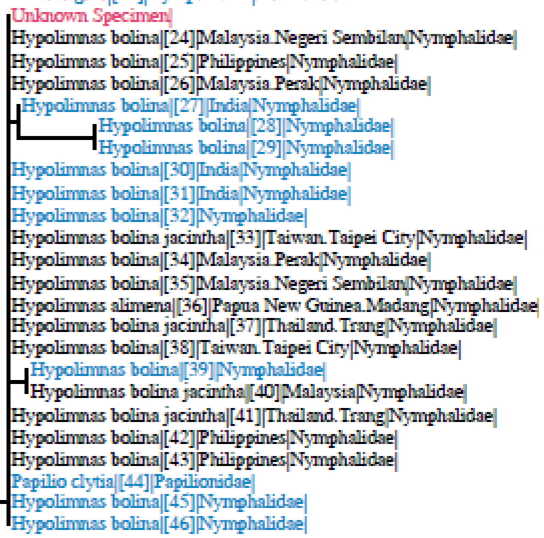

Tree 39 – 2 multi exclusive

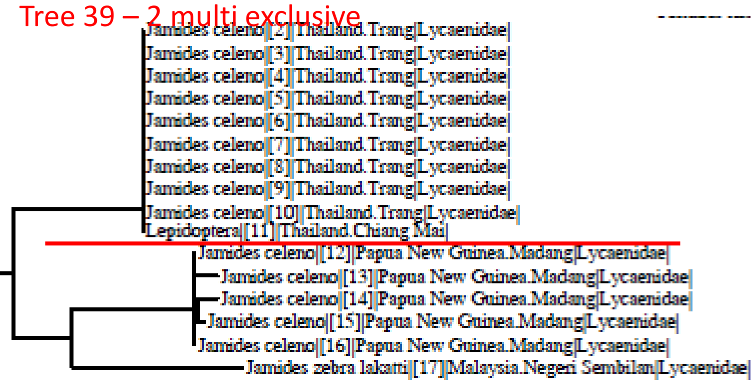

Tree 40 – 2 multi identical

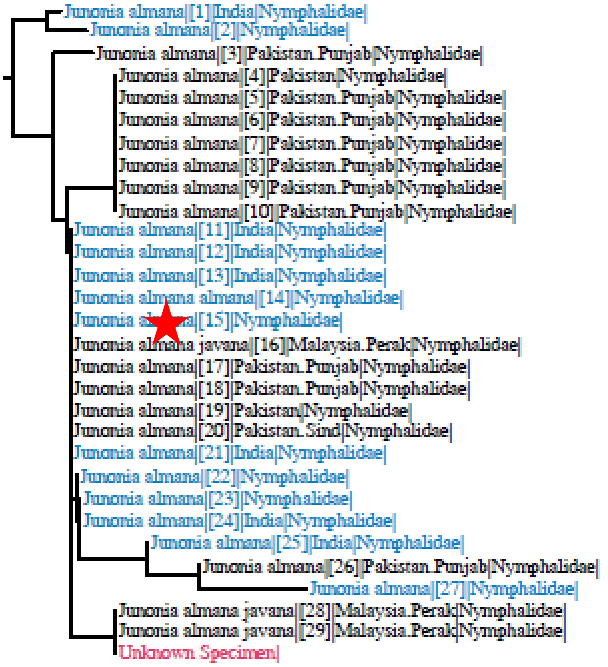

Tree 41 – 1 singleton unique; 1 multi unique; 1 multi exclusive

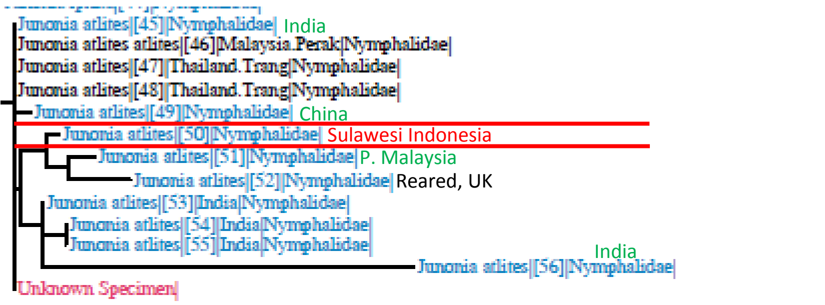

Tree 42 – 1 singleton unique; 1 multi exclusive

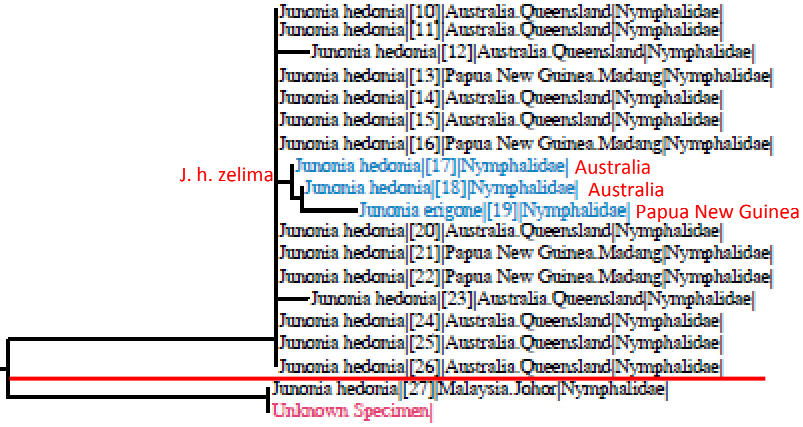

Tree 43 – 2 multi exclusive

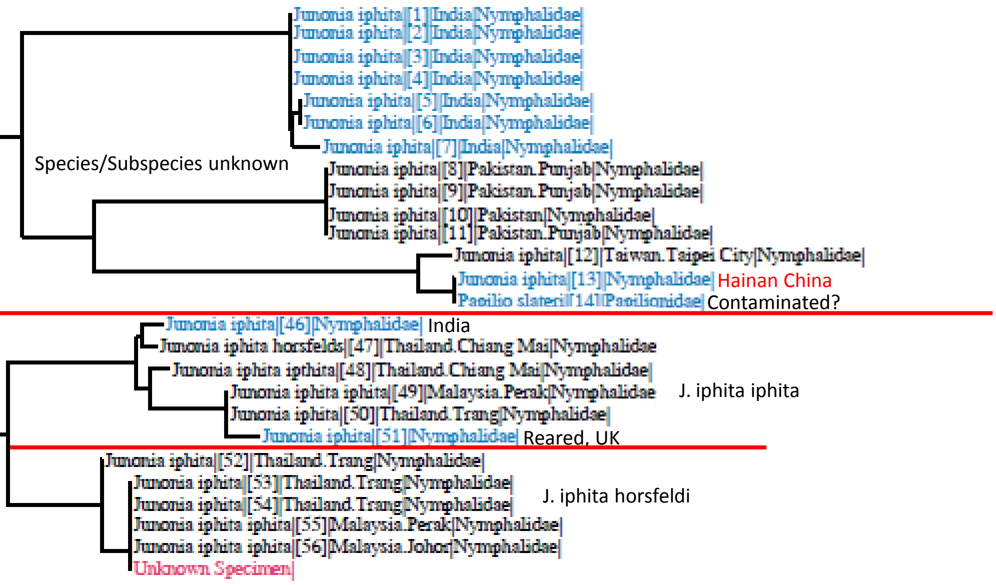

Tree 44 – 2 multi identical; 1 multi exclusive

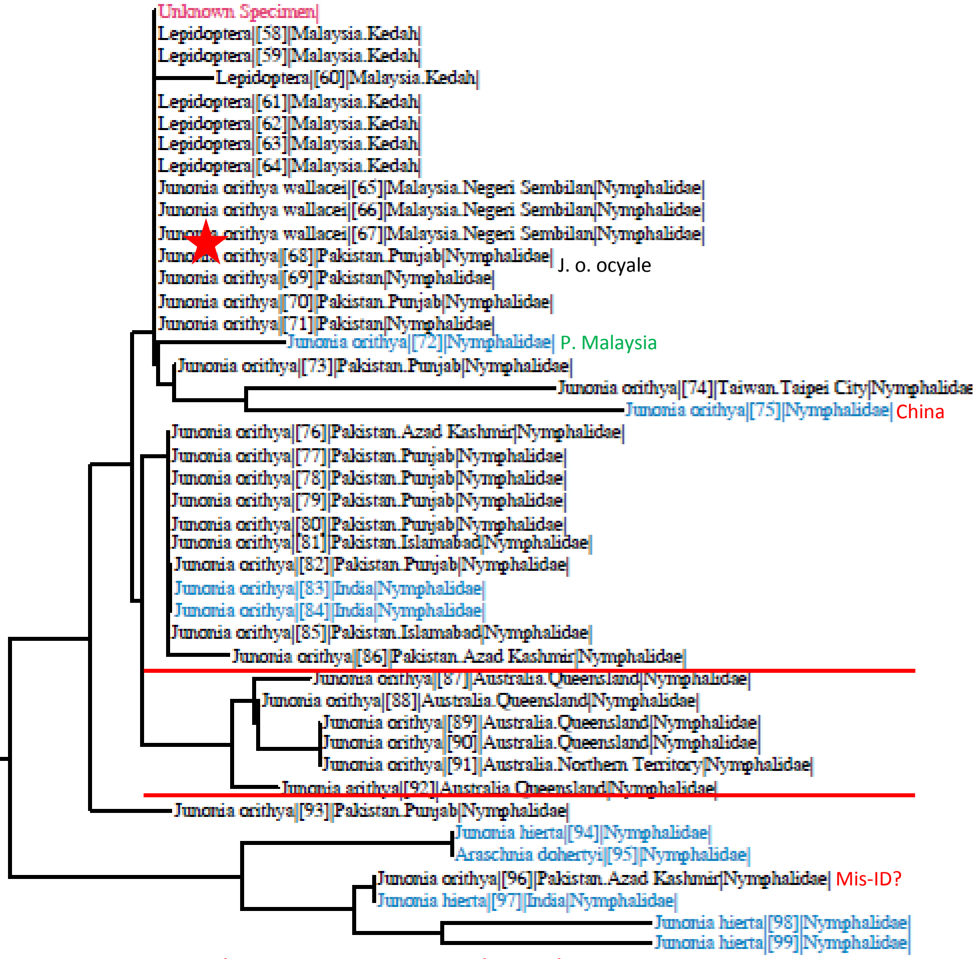

Tree 45 – 3 singletons unique; 1 multi exclusive

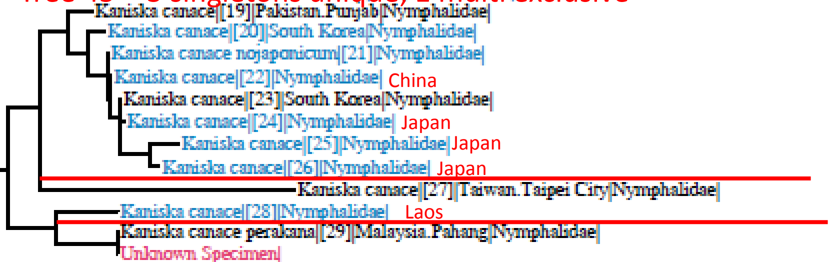

Tree 46 – 1 singleton unique; 1 multi exclusive

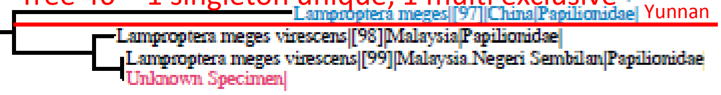

Tree 47 – 2 singleton unique

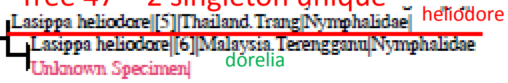

Tree 48 – 1 singleton unique; 1 multi exclusive

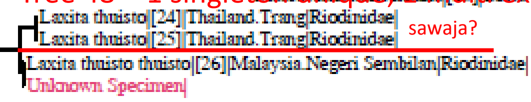

Tree 49 – 1 singleton identical; 1 multi identical

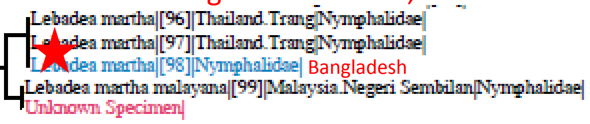

Tree 50 – 1 multi unique; 1 multi exclusive

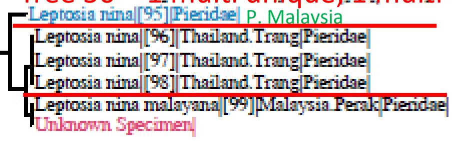

Tree 51 – 1 singleton unique; 1 multi exclusive

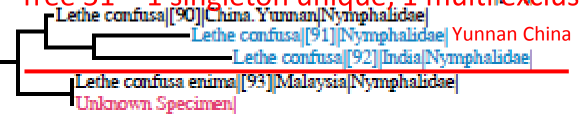

Tree 52 – 2 multi exclusive

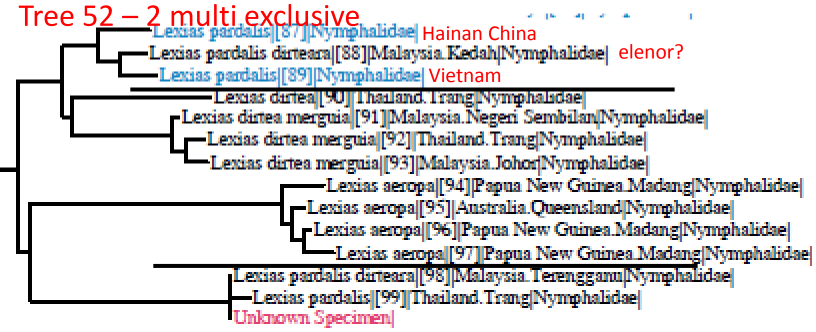

Tree 53 – 2 multi identical

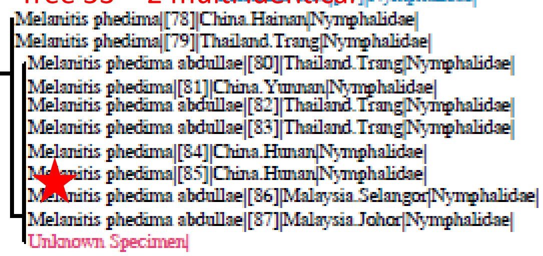

Tree 54 – 1 singleton unique; 1 multi exclusive

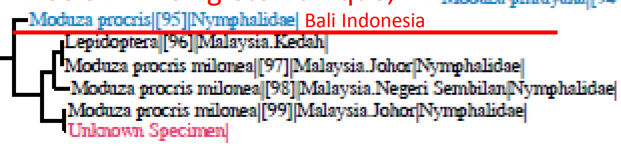

Tree 55 – 1 singleton unique; 1 multi exclusive

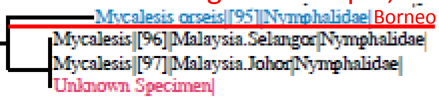

Tree 56 – 2 singleton unique

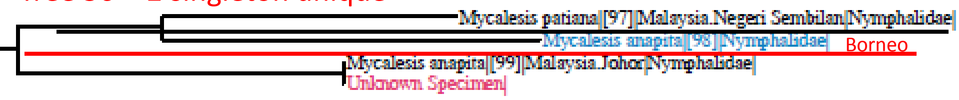

Tree 57 – 2 singleton identical

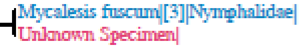

Tree 58 – 2 singleton unique

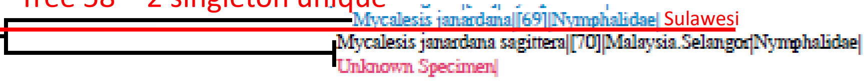

Tree 59 – 1 singleton unique; 1 multi unique, 2 multi exclusive

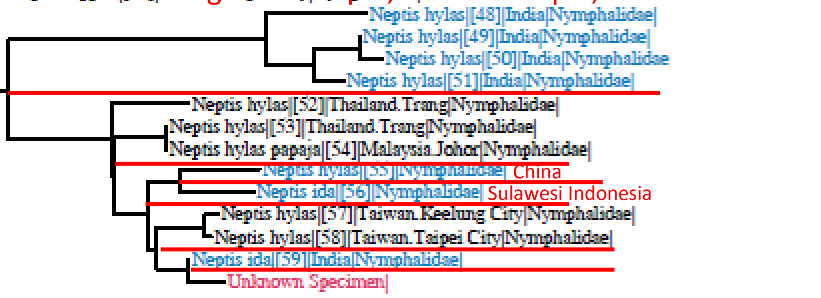

Tree 60 – 2 multi exclusive

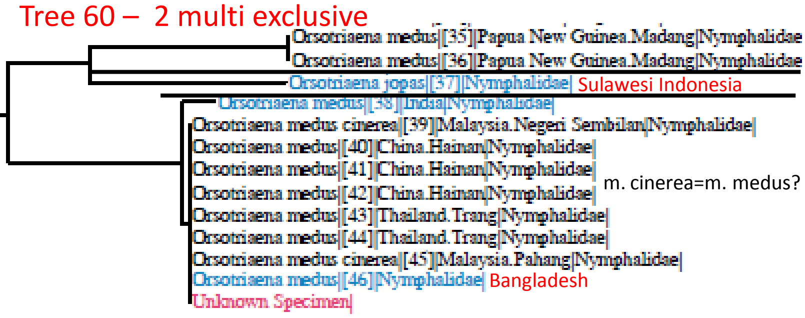

Tree 61 – 1 singleton unique; 1 multi unique

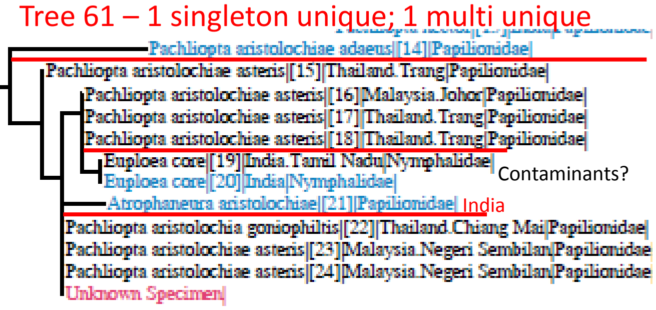

Tree 62 – 3 singletons unique; 1 multi exclusive

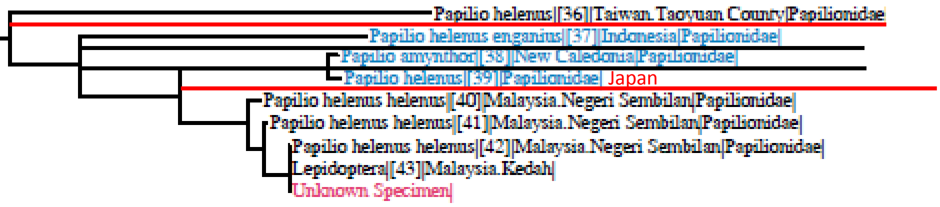

Tree 63 – 1 singleton identical; 1 singleton unique; 1 multi identical; 1 multi exclusive

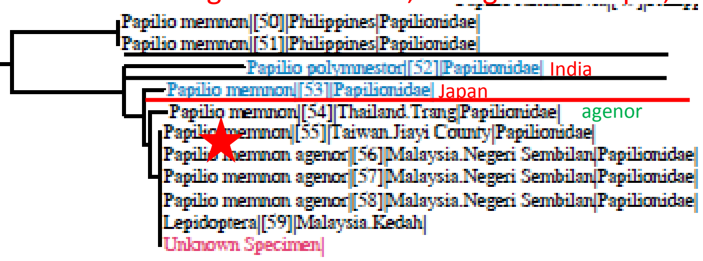

Tree 64 – 2 multi exclusive

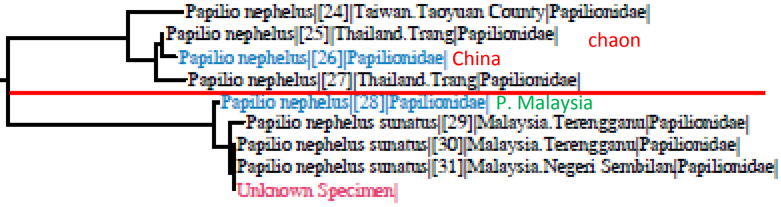

Tree 65 – 1 singleton unique; 2 multi identical

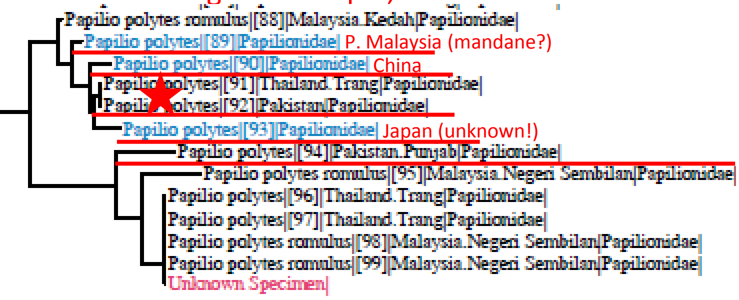

Tree 66 – 1 singleton unique; 1 multi unique

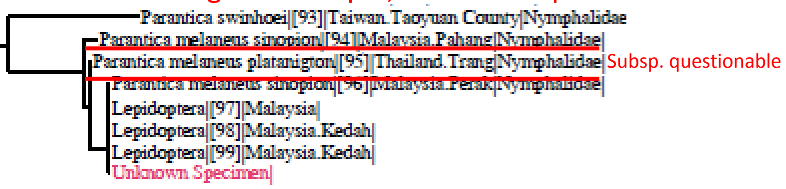

Tree 67 – 2 singleton unique; 1 multi exclusive

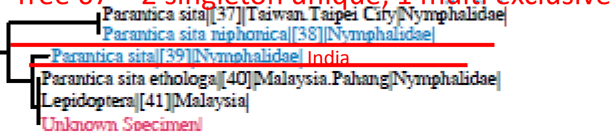

Tree 68 – 2 singleton unique

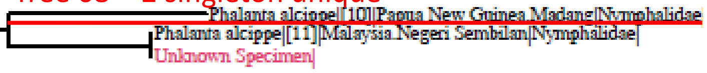

Tree 69 – 1 singleton unique; 2 multi exclusive

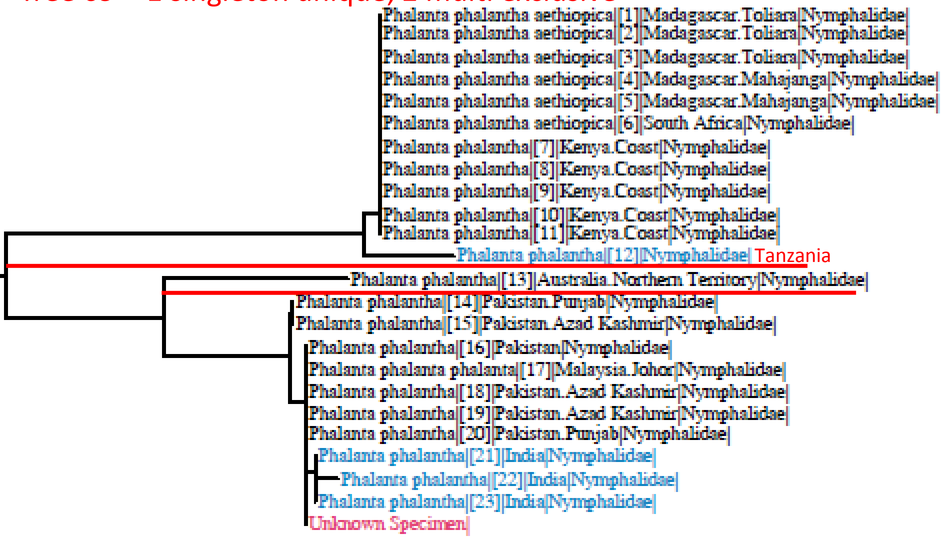

Tree 70 – 1 singleton unique, 1 multi unique

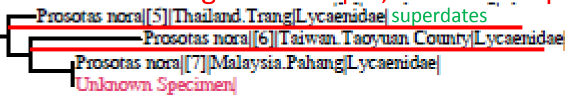

Tree 71 – 2 singleton unique

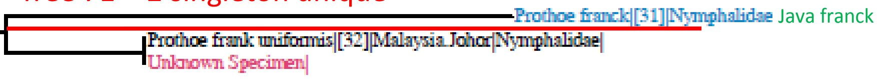

Tree 72 – 2 multi unique

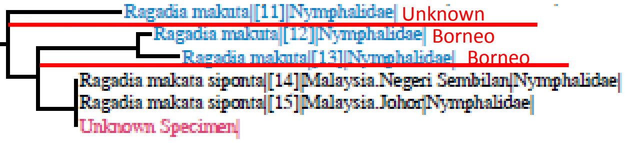

Tree 73 – 2 singleton unique

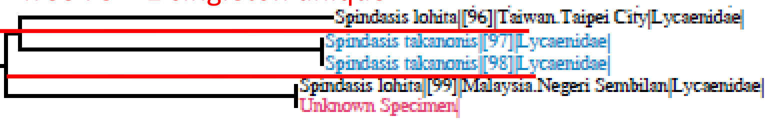

Tree 74 – 1 singleton unique; 1 multi exclusive

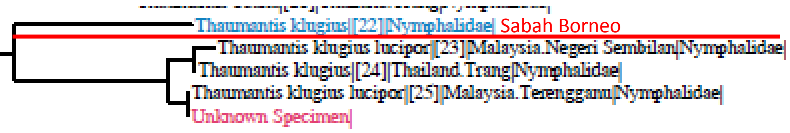

Tree 75 – 2 multi exclusive

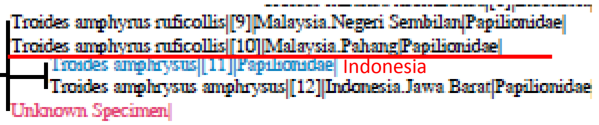

Tree 76 – 1 singleton identical; 1 multi identical

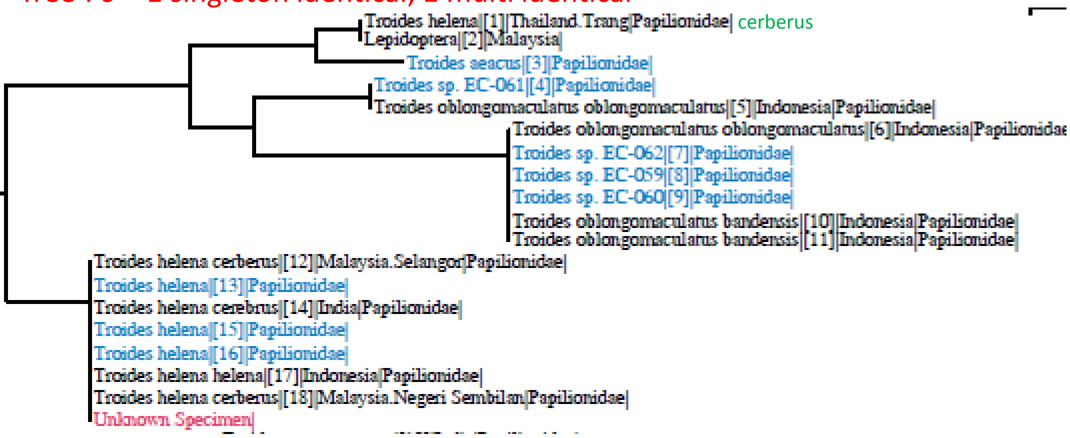

Tree 77 – 1 singleton unique; 1 multi exclusive

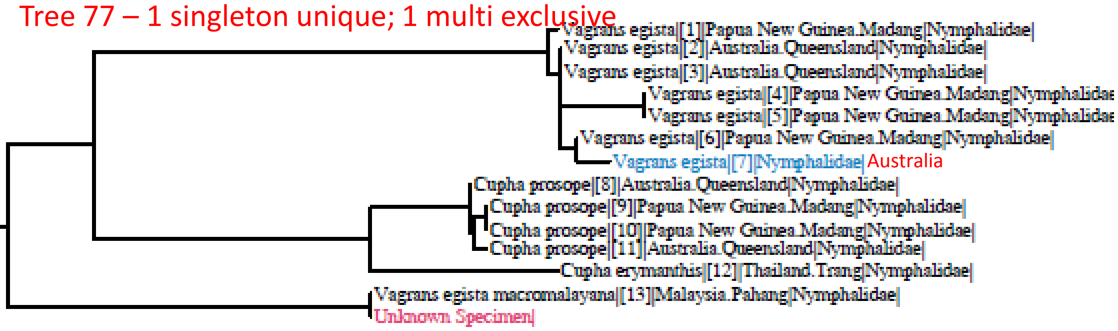

Tree 78 – 1 singleton unique; 1 mutli exclusive

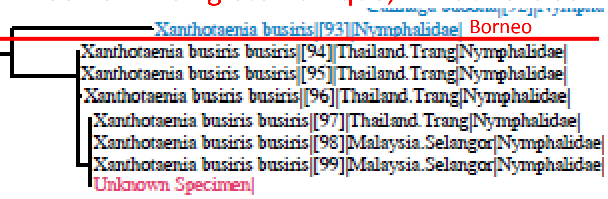

Tree 79 – 2 multi exclusive

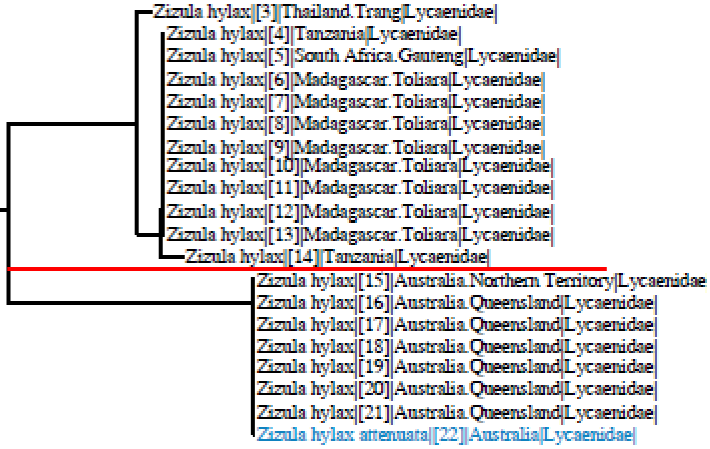

Supplement: Subspecies Trees S1 — Trees resulting from a BOLD tree based identification of UMKL DNA barcodes (PDF) [file pone.0079969.s007.pdf]
